# The inhibitory effect and mechanism of Resina Draconis on the proliferation of MCF-7 breast cancer cells: a network pharmacology-based analysis

**DOI:** 10.1038/s41598-023-30585-0

**Published:** 2023-03-07

**Authors:** Yana Lv, Yan Mou, Jing Su, Shifang Liu, Xuan Ding, Yin Yuan, Ge Li, Guang Li

**Affiliations:** 1 Yunnan Branch of the Institute of Medicinal Plant Development, Chinese Academy of Medical Sciences, Jinghong, 666100 China; 2 Yunnan Key Laboratory of Southern Medicinal Utilization, Jinghong, 666100 China; 3 Yuxi Normal University, Yuxi, 653100 China

**Keywords:** Breast cancer, Target identification, Drug discovery

## Abstract

Resina Draconis (RD) is known as the "holy medicine for promoting blood circulation" and possesses antitumor properties against various types of cancer, including breast cancer (BC); however, the underlying mechanism is not well understood. To explore the potential mechanism of RD against BC using network pharmacology and experimental validation, data on bioactive compounds, potential targets of RD, and related genes of BC were obtained from multiple public databases. Gene Ontology (GO) and KEGG pathway analyses were performed via the DAVID database. Protein interactions were downloaded from the STRING database. The mRNA and protein expression levels and survival analysis of the hub targets were analyzed using the UALCAN, HPA, Kaplan‒Meier mapper, and cBioPortal databases. Subsequently, molecular docking was used to verify the selected key ingredients and hub targets. Finally, the predicted results of network pharmacology methods were verified by cell experiments. In total, 160 active ingredients were obtained, and 148 RD target genes for the treatment of BC were identified. KEGG pathway analysis indicated that RD exerted its therapeutic effects on BC by regulating multiple pathways. Of these, the PI3K-AKT pathway was indicated to play an important role. In addition, RD treatment of BC seemed to involve the regulation of hub targets that were identified based on PPI interaction network analysis. Validation in different databases showed that AKT1, ESR1, HSP90AA1, CASP3, SRC and MDM2 may be involved in the carcinogenesis and progression of BC and that ESR1, IGF1 and HSP90AA1 were correlated with worse overall survival (OS) in BC patients. Molecular docking results showed that 103 active compounds have good binding activity with the hub targets, among which flavonoid compounds were the most important active components. Therefore, the sanguis draconis flavones (SDF) were selected for subsequent cell experiments. The experimental results showed that SDF significantly inhibited the cell cycle and cell proliferation of MCF-7 cells through the PI3K/AKT pathway and induced MCF-7 cell apoptosis. This study has preliminarily reported on the active ingredients, potential targets, and molecular mechanism of RD against BC, and RD was shown to exert its therapeutic effects on BC by regulating the PI3K/AKT pathway and related gene targets. Importantly, our work could provide a theoretical basis for further study of the complex anti-BC mechanism of RD.

## Introduction

Breast cancer (BC) is one of the most common malignant tumors in the clinic and has become the most common malignancy among women worldwide; although the disease survival rate has improved, the 5-year survival rate of patients with metastatic BC is less than 30%^1^. The most recent global cancer burden figures estimated that there were 2.3 million (11.7%) new BC cases and 684,996 (6.9%) cancer deaths in 2020^2^. Basically, the high mortality of patients with BC is attributed to high heterogeneity and molecular complexity^3^. Currently, surgical resection, radiotherapy, chemotherapy, hormone therapy and targeted therapy are still the most common clinical treatment methods for BC^4,5^. However, not every patient with metastatic or advanced BC is fit for these therapies, and some patients respond poorly to these treatments. Surgical resection does not improve prognosis, and postoperative infection may cause serious complications and even threaten the survival of patients, while long-term radiotherapy and chemotherapy can trigger many adverse reactions involving hair loss, nausea, anorexia, premature ovarian failure, and cardiac dysfunction, thus reducing a patient's quality of life^6,7^. In addition, most anticancer drugs have side effects and multidrug resistance and lack of tumor selectivity are substantial challenges^8^. Therefore, it is urgent to seek natural drugs that are less toxic, associated with fewer side effects and safer for the treatment of BC.

Resina Draconis (RD) (Chinese name: Longxuejie) is derived from the red resins of *Dracaena cochinchinensis* (Lour.) S.C. Chen, which grows mainly in the Chinese provinces of Guangxi, Yunnan and Hainan^9^. RD, known as the "holy medicine for promoting blood circulation", is a rare and precious traditional medicine in China that has a variety of effects, including activating blood to remove blood stasis, hemostasia, acesodyne, myogenic, and sore healing^9–12^. Therefore, it is clinically used to treat cardiovascular and cerebrovascular diseases, gynecopathy, blood stasis syndrome and trauma. Phytochemical studies have shown that RD mainly contains flavonoids, terpenes, phenols, steroids, and other types of compounds, of which flavonoids are the main chemical constituents^13–17^. It has been reported that the extracts and compounds of RD have antithrombotic, anti-inflammatory, antidiabetic, antibacterial, antispasmodic, analgesic, antiulcer and antitumor effects. Several studies have shown that RD exerts antitumor effects mainly by inhibiting cell proliferation, inducing apoptosis, cell cycle arrest, DNA damage, and autophagy, and repressing metastasis and angiogenesis^18–23^. Loureirin A is the primary chemical component of RD that can trigger apoptosis and inhibit the growth of breast tumor cells (MCF-7 cells)^24^. Resveratrol inhibits the migration and metastasis of breast tumor cells (MDA-MB-231) by reversing TGF-β1-induced epithelial-mesenchymal transition, as shown by Lee et al^22^. Liang et al. reported that liquiritigenin can inhibit the proliferation and induce apoptosis of triple-negative BC cells^25^. Although studies have confirmed the anti-BC activity of certain active components of RD, the active ingredients and targets remain unclear, and the underlying molecular mechanisms are poorly understood.

Due to the complexity of traditional Chinese medicine (TCM) constituents and the interaction between Chinese medicine and the human body, it is difficult to understand the molecular mechanism because of synergistic effects on multiple components that can interact with multiple targets simultaneously^26^. Network pharmacology can quickly and effectively assess TCM because its research strategy is consistent with the holistic and systemic views of TCM. The most important feature of network pharmacology is predictability, which emphasizes the multichannel regulation of signaling pathways, improves the therapeutic effect of drugs, and reduces toxicity and side effects^27^. TCM network pharmacology can provide a potential strategy for deciphering the molecular mechanisms of TCM treatments for various diseases and determining their active ingredients or combinations from the perspective of biological networks^28^. In this study, we first attempted to apply a network pharmacology approach to explore the main bioactive components of RD and predict hub genes and pathways involved in the pathogenesis of RD against BC. In addition, molecular docking was used to simulate the binding ability of the main bioactive components with core targets. Finally, cell experimental studies were adopted to uncover the pharmacodynamic effects and mechanisms of RD against BC.

## Results

### Bioactive compound screening

In total, 221 compounds were retrieved from the literature, namely, 144 flavonoid compounds, 23 phenolic compounds, 37 steroid compounds and 17 other compounds (Supplementary Table S1). After identifying the relevant structures, 115 bioactive compounds were screened by using SwissADME with GI and DL, which included 87 flavonoid compounds, 22 phenolic compounds and 6 steroid compounds. A total of 378 potential targets of the bioactive compounds were obtained by using the PharmMapper database (Supplementary Table S2).

### Mining RD target genes for the treatment of BC

**Figure 1:**
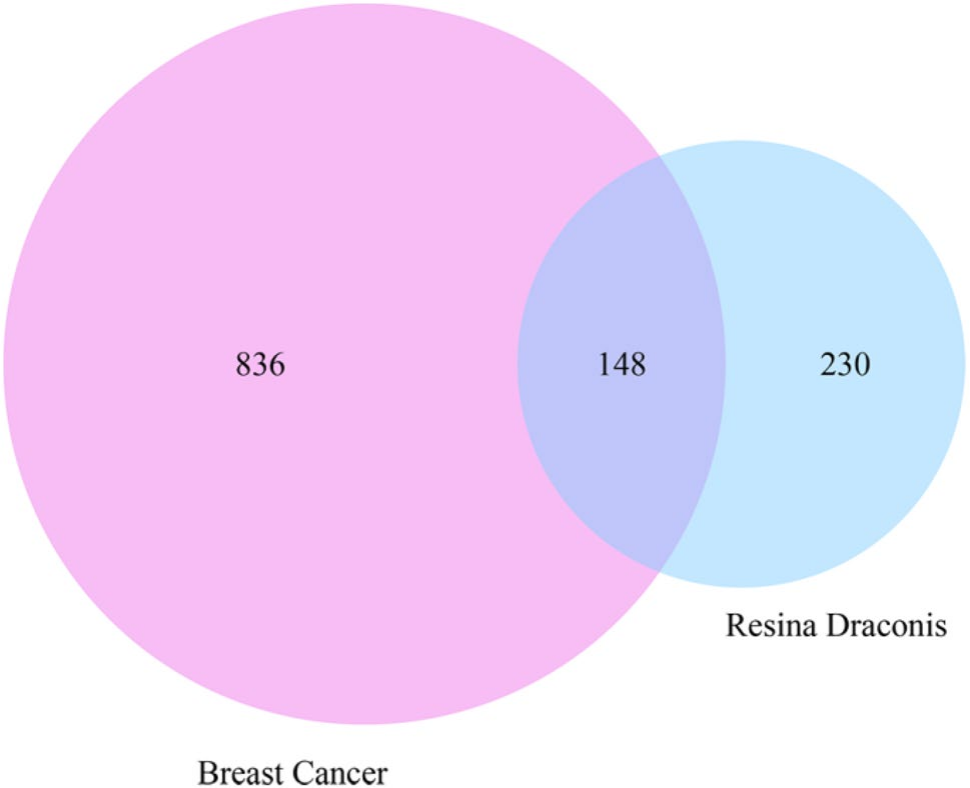
Venn diagram of the potential targets of RD against BC.

A total of 984 BC-related genes were identified by merging the genes retrieved from the DisGeNET, GeneCards, OMIM, PharmGKB, and TTD databases and removing duplicates (Supplementary Table S3). Furthermore, 148 RD target genes for the treatment of BC were obtained from common targets of both BC and the chemical constituents of RD (Fig. 1, Supplementary Table S4).

### GO and KEGG enrichment analysis

**Figure 2:**
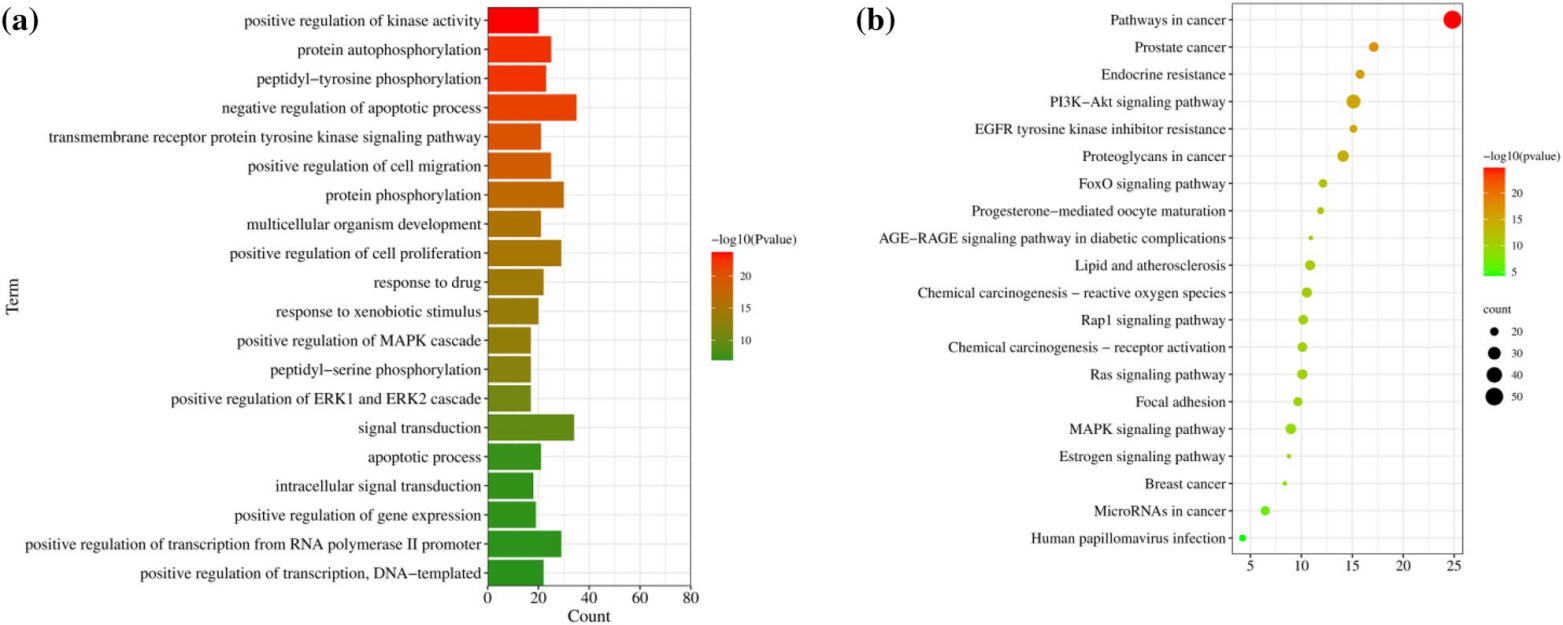
Top 20 GO BP terms and KEGG pathways. (**a**) BP enrichment. (**b**) KEGG pathway enrichment. The color of the bar chart represents the adjusted p value.

To reveal the biological function and the potential mechanism underlying RD against BC, GO functional and KEGG pathway analyses were performed using the DAVID database. A total of 227 biological process (BP) term were identified (Supplementary Table S5). The top 20 BP terms are shown in Fig. 2a. Terms related to BP mainly focused on the negative regulation of apoptotic process, signal transduction, protein phosphorylation, and positive regulation of cell proliferation. In addition, 142 KEGG pathways were identified (Supplementary Table S6). The top 20 KEGG signaling pathways are shown in a bubble diagram (Fig. 2b), which mainly includes pathways in cancer, the PI3K-Akt signaling pathway, the MAPK signaling pathway, the Ras signaling pathway, and the Rap1 signaling pathway.

### Construction of the compound-target-pathway network

**Figure 3:**
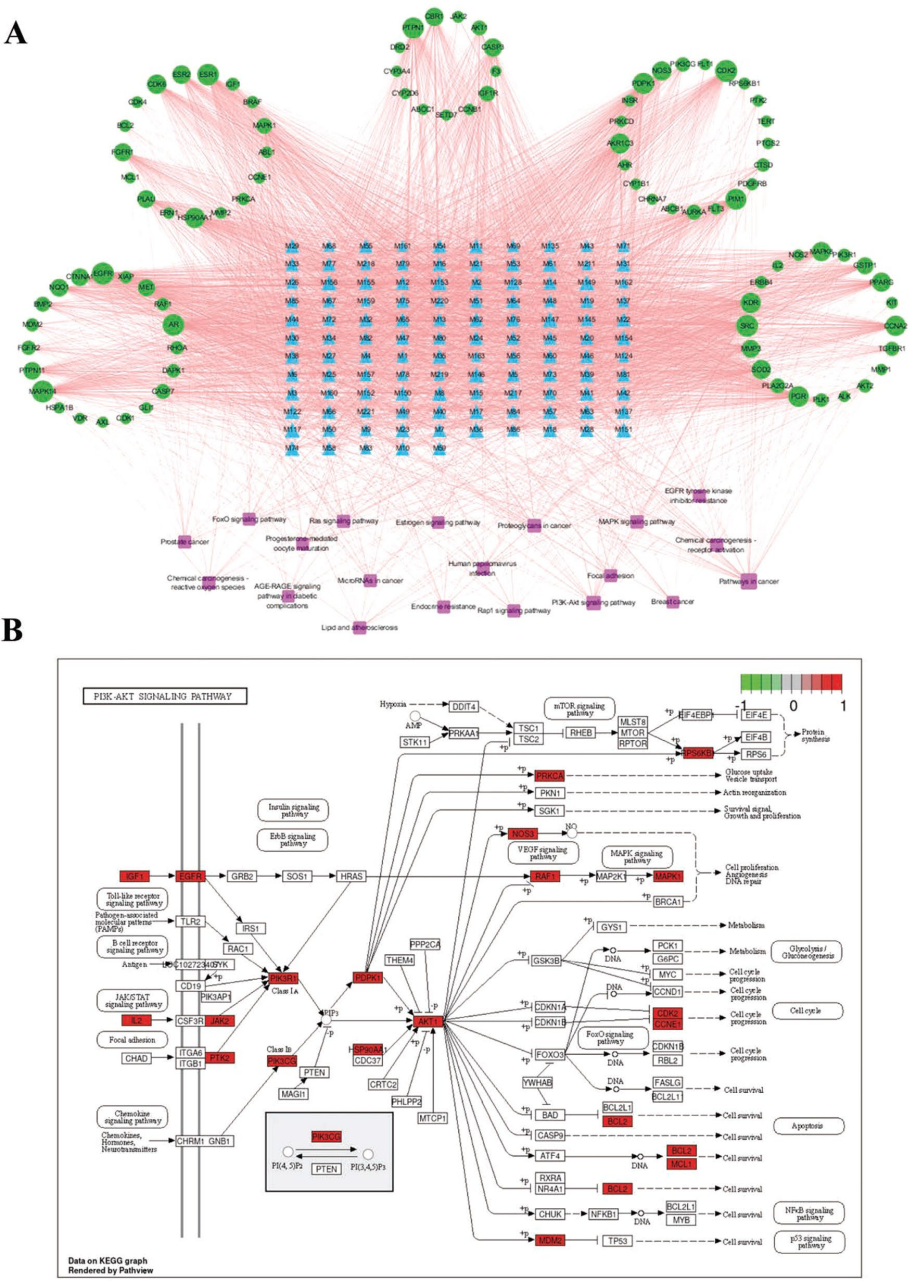
The component-target-pathway network and PI3K-AKT signaling pathway. (**a**) The component-target-pathway network. Green circles represent targets, blue triangles represent compounds, and pink squares represent pathways. Node sizes are proportional to their degree. (**b**) PI3K-AKT signaling pathway. The pink rectangle represents the key targets.

Based on the KEGG pathway enrichment results, the top 20 pathways and 91 enriched genes corresponding to 115 compounds were selected to construct the compound-target-pathway network, which included 226 nodes and 3280 edges (Fig. 3a, Supplementary Table S7). According to the network analysis, a single compound affected multiple targets that participated in multiple pathways; in turn, multiple compounds were closely related to unique targets and unique pathways. The results revealed that the processes of RD for the treatment of BC involve multiple components, multiple targets, and multiple pathways. In addition, among the BC-related signaling pathways, the PI3K-AKT signaling pathway^29^, which had the second most enriched genes, could play an important role in the treatment of BC (Fig. 3b).

### Construction of the PPI network and screening of hub targets

**Figure 4:**
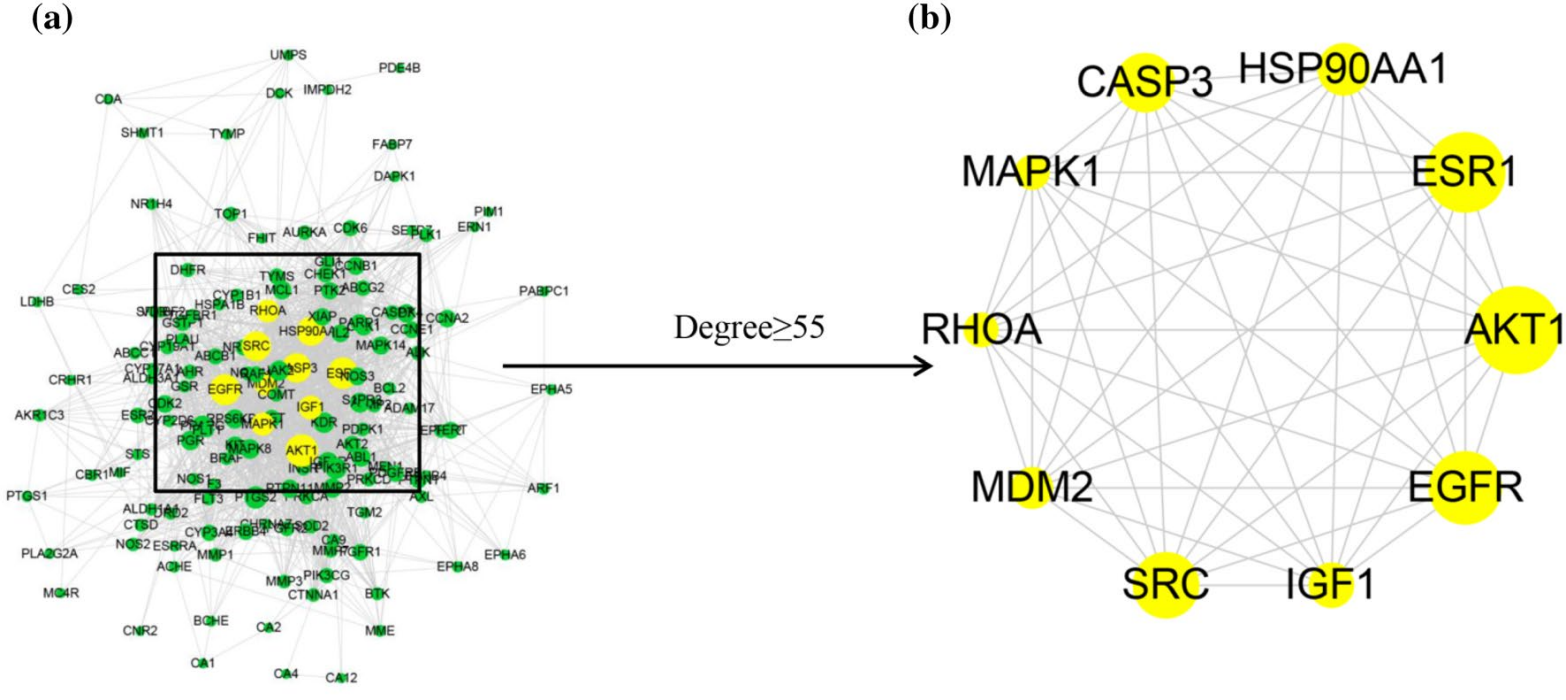
The PPI network and 10 hub targets. (**a**) The PPI network visualized by Cytoscape. (**b**) Ten hub targets identified by cytoHubba. The larger the node is, the more important the target in the network.

To identify biomarkers of RD treatment for BC, 148 RD target genes were imported into the STRING database to obtain protein–protein interaction (PPI) information. Next, a PPI network that included 146 nodes and 1668 edges was visualized with Cytoscape v3.6.1 (Fig. 4a). In addition, cytoHubba was used to construct a core PPI subnetwork composed of 10 genes with the highest degree values. Finally, the top 10 hub targets were precisely aligned as AKT1, ESR1, EGFR, SRC, CASP3, HSP90AA1, IGF1, MDM2, RHOA, and MAPK1 (Fig. 4b and Table 1).

**Table 1:** The hub targets of RD in BC treatment and the topological parameters.

Number	Gene name	Protein name	Degree
1	AKT1	RAC-alpha serine/threonine-protein kinase	94
2	ESR1	Estrogen receptor 1	88
3	EGFR	Epidermal growth factor receptor	87
4	SRC	Proto-oncogene tyrosine-protein kinase Src	82
5	CASP3	Caspase-3	80
6	HSP90AA1	Heat shock protein 90 alpha family class A member 1	79
7	IGF1	Insulin-like growth factor 1	59
8	MDM2	MDM2 proto-oncogene	56
9	RHOA	Ras homolog family member A	55
10	MAPK1	Matrix metalloproteinase-1	55

### Validation of hub targets in different databases

**Figure 5:**
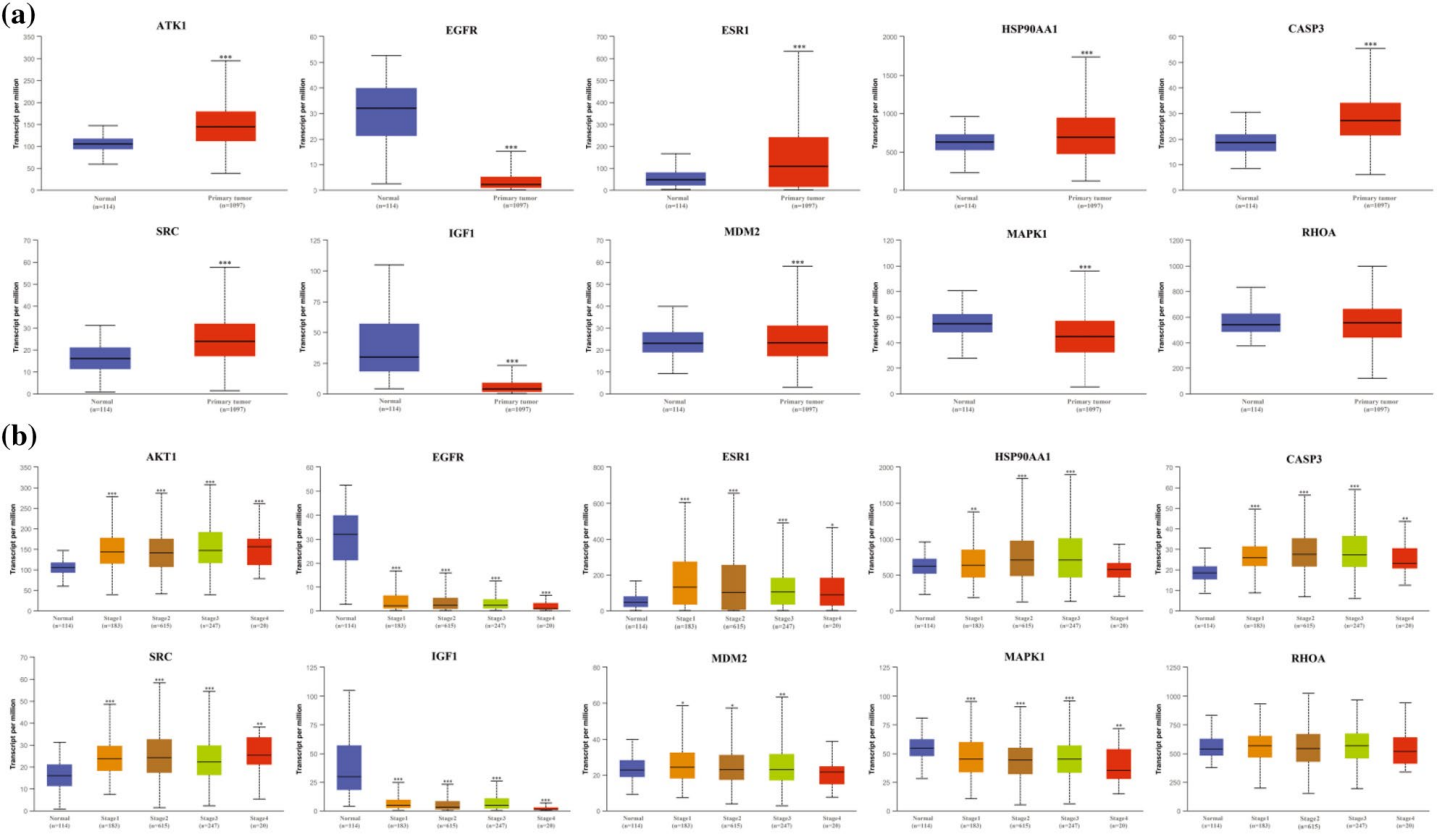
Validation of the mRNA expression of hub targets in UALCAN. (**a**) The mRNA levels of 10 hub targets in BC tissues and normal tissues (***P < 0.001). (**b**) The mRNA levels of 10 hub targets in different tumor stages of BC (*P < 0.05; **P < 0.01; ***P < 0.001).

To elucidate the possible therapeutic significance and prognostic value of the 10 hub targets, the mRNA expression and protein expression of the molecules were analyzed. In the cases of invasive BC from the TCGA database in UALCAN (Fig. 5a), the mRNA levels of AKT1, ESR1, HSP90AA1, CASP3, SRC, and MDM2 were significantly increased in tumor tissues. In turn, EGFR, IGF1 and MAPK1 mRNA expression levels were significantly decreased, while RHOA mRNA expression was not significantly downregulated in tumor tissues.

Next, we investigated the correlation between the mRNA levels of 10 hub targets and different tumor stages of BC in the UALCAN database. As shown in Fig. 5b, when compared with normal levels, the mRNA levels of EGFR, IGF1 and MAPK1 were decreased significantly in stages one to four BC tissues, while the expression levels of AKT1, ESR1, HSP90AA1, CASP3, SRC, and MDM2 were increased significantly, with the exception of the levels of HSP90AA1 and MDM2, which were not significantly increased in stage four BC tissues. In addition, the expression of RHOA in different tumor stages had different degrees of expression than that in normal tissues, but the difference was not significant.

**Figure 6:**
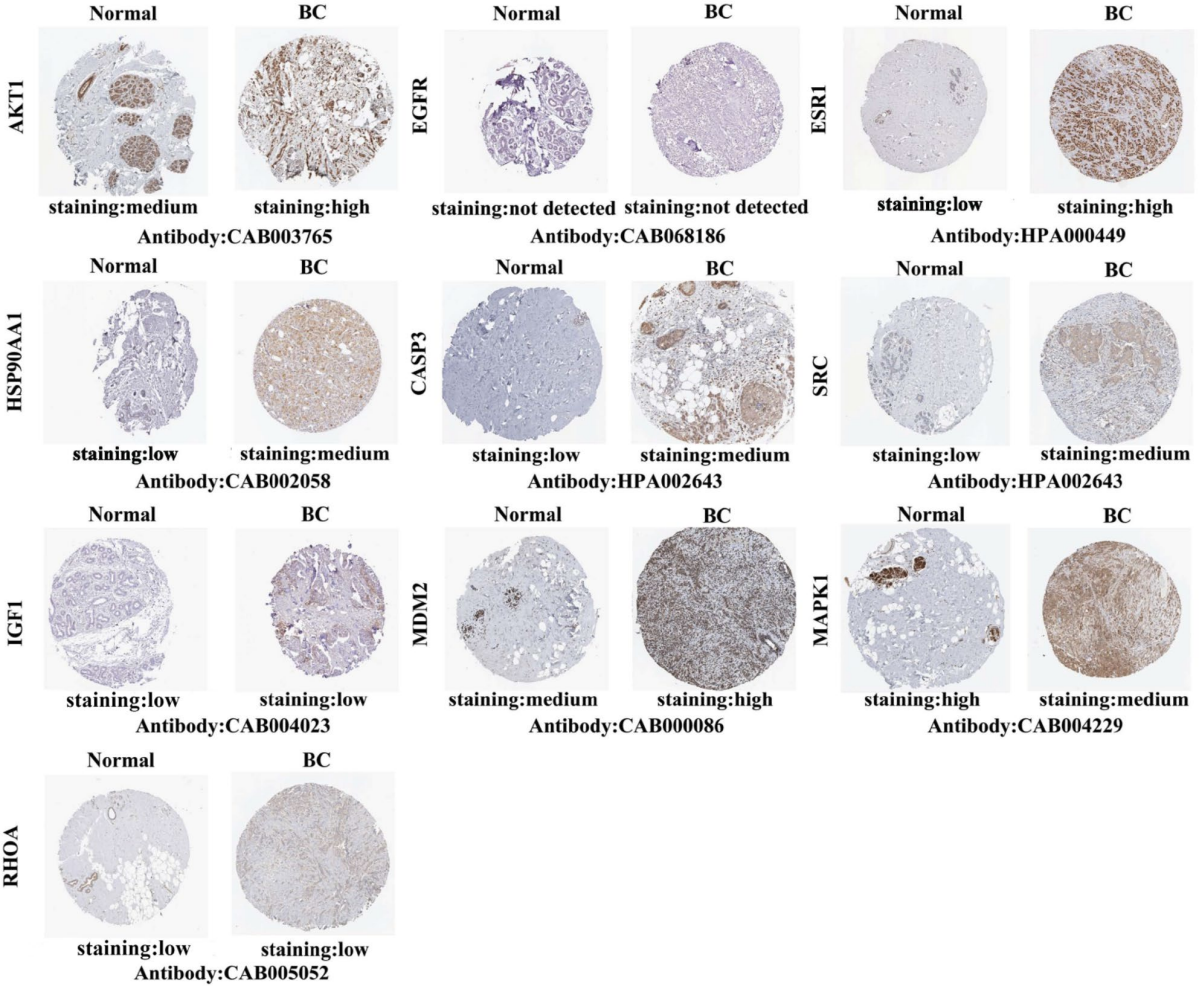
Immunohistochemical images of 10 hub targets in normal breast tissues and BC tissues (HPA Database).

We further studied the protein levels of 10 hub targets in BC tissues. As shown in Fig. 6, the protein expression of EGFR, IGF1 and RHOA was not different between normal breast tissues and BC tissues. In turn, the other 7 hub targets were expressed to different degrees between normal breast tissues and BC tissues, indicating that there were significant differences. Compared with normal breast tissues, the expression levels of AKT1, ESR1, HSP90AA1, CASP3, SRC, and MDM2 were increased in BC tissues, while the expression levels of MAPK1 were decreased in BC tissues. These results indicated that aberrant expression of several hub targets occurs at the protein level in patients with BC.

**Figure 7:**
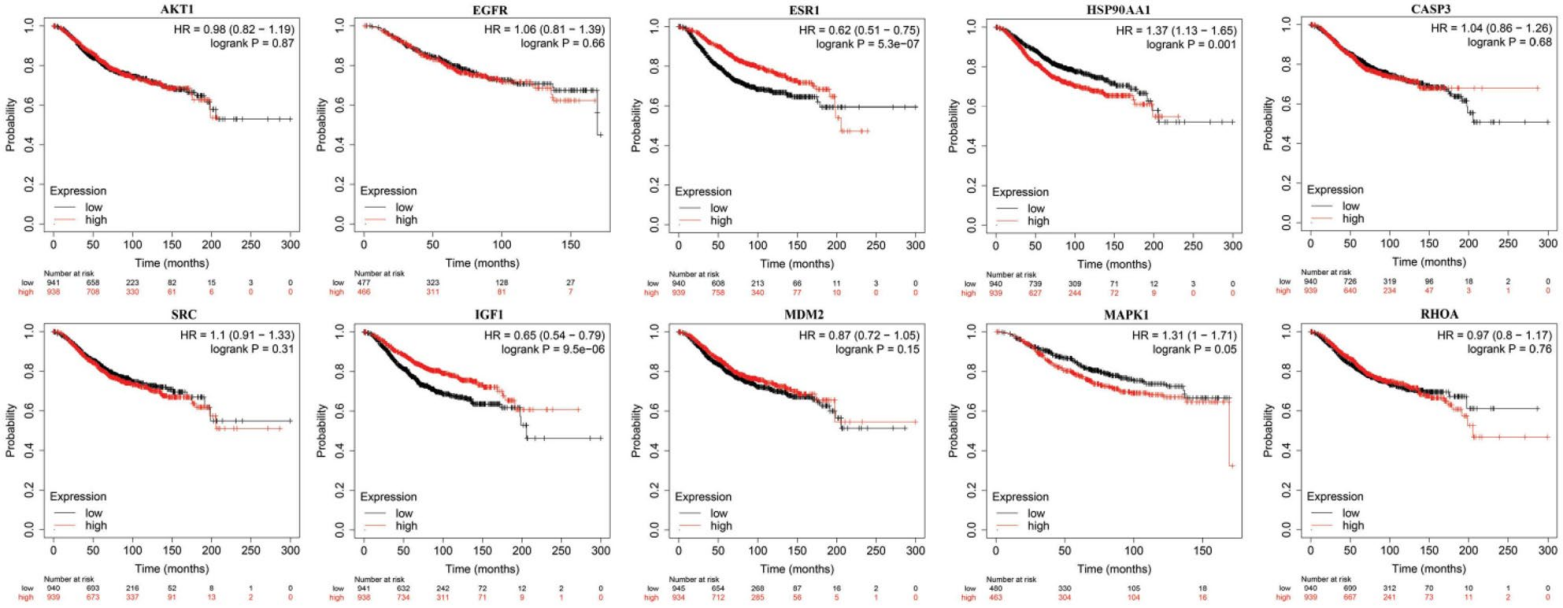
OS analysis of 10 hub targets in BC (Kaplan–Meier plot).

We next explored the prognostic significance of 10 hub targets in BC patients using the Kaplan‒Meier mapper database. As shown in Fig. 7, of the 10 genes, we found that ESR1 (hazard ratio [HR] = 0.62, log-rank P = 5.3e−07), HSP90AA1 (HR = 1.37, log-rank P = 0.001), and IGF1 (HR = 0.65, log-rank P = 9.5e−06) were significantly correlated with OS. In contrast, AKT1 (HR = 096, log-rank P = 0.87), EGFR (HR = 1.1, log-rank P = 0.66), CASP3 (HR = 1.04, log-rank P = 0.68), SRC (HR = 0.97, log-rank P = 0.31), MDM2 (HR = 1.05, log-rank P = 0.15), MAPK1 (HR = 1.31, log-rank P = 0.05) and RHOA (HR = 1.37, log-rank P = 0.76) were not significantly associated with OS in BC patients. The results indicated that low expression of ESR1 and IGF1 and high expression of HSP90AA1 were associated with significantly poor survival.

**Figure 8:**
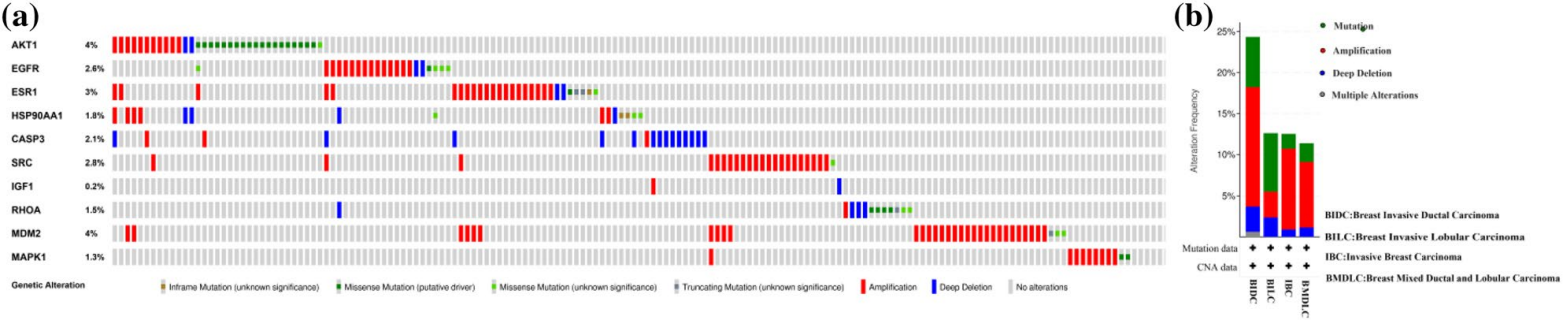
Genetic alterations in 10 hub targets in BC patients (cBioPortal). (**a**) OncoPrint visual summary of genetic alterations detected in 10 hub targets. (**b**) Summary of alterations in 10 hub targets in different BC types.

We next analyzed the frequency and types of gene changes in 10 hub targets in 818 BC patients using the cBioPortal tool. As shown in Fig. 8a, the overall alteration rate of 10 hub targets was 19% (159/818), and the genetic alteration rate of individual genes varied from 0.2 to 4%, of which the variation rate of AKT1 and MDM2 was the highest (4%) and the variation rate of IGF1 was the lowest (0.2%). In addition, among the 4 different BC types, the genetic alteration rate ranged from 11.36 to 20.34% (Fig. 8b). These results indicated that the types of genetic variation in 10 hub targets included gene mutation, gene amplification, deep deletion and multiple alterations, of which the mutation type in AKT1, EGFR, ESR1, SRC, and MDM2 was mainly gene amplification.

### Molecular docking validation

**Figure 9:**
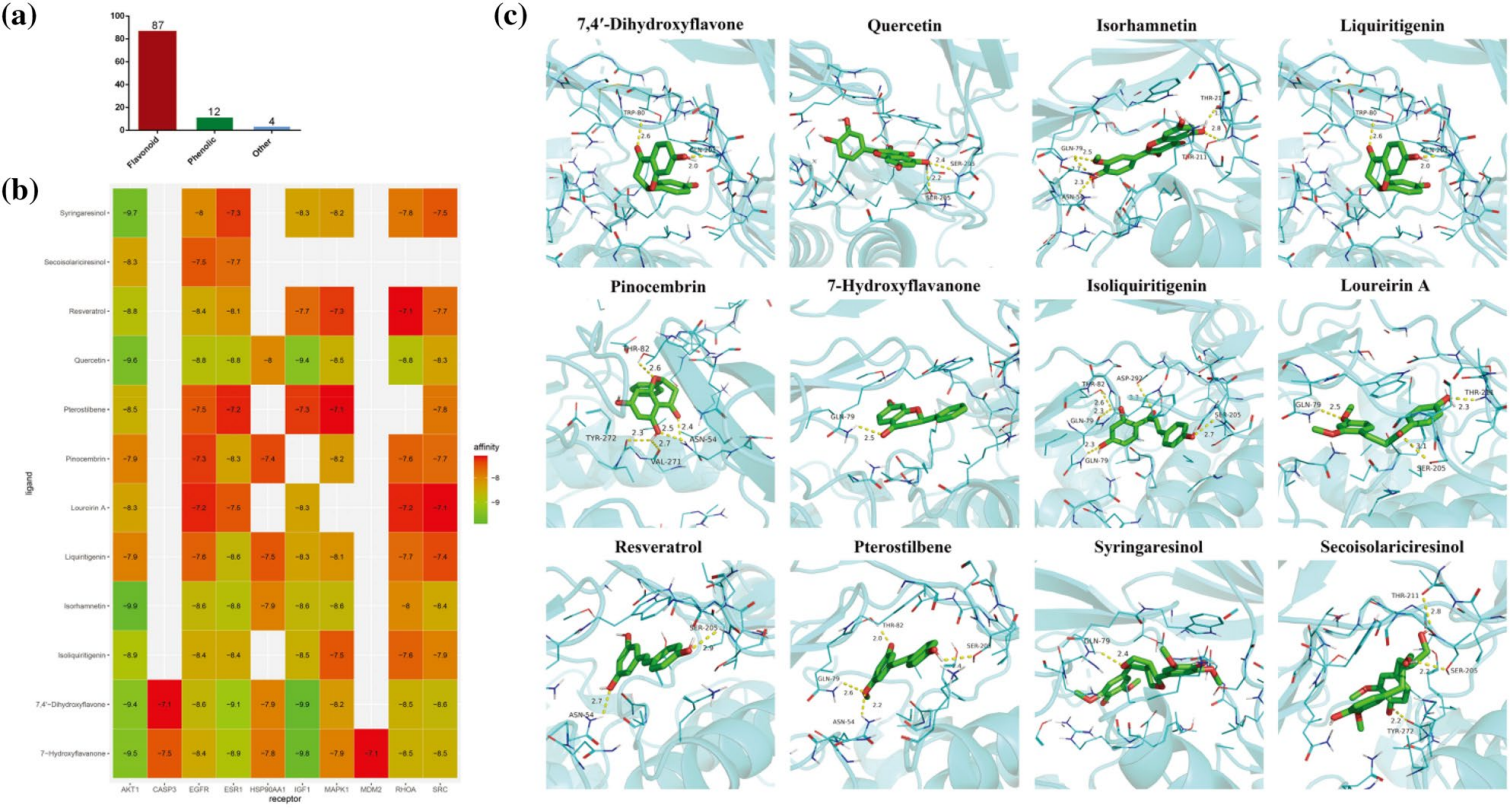
Molecular docking results. (**a**) The proportion of different types of compound docking scores <  − 7.0. Molecular docking results. (**b**) Heatmap of binding between 10 hub targets and 12 active components. The greener the color is, the better the binding activity. (**c**) Molecular docking models between 12 active compounds and AKT1. The green sticks represent the ligand, yellow dashed lines represent the hydrogen bonds, and the number represents the binding distance.

To further validate and explore the mode of interaction between target genes and effective compounds, 10 hub targets and their corresponding 108 compounds were selected to perform molecular docking. In total, 103 compounds were ultimately screened with docking scores < − 7.0 to 10, which indicated that they had strong binding activity (Supplementary Table S8). As shown in Fig. 9a, the largest proportion (84.5%) of flavonoid compounds suggested that flavonoids are the main ingredient in the anti-BC action of RD, while phenolic (11.7%) and other compounds (3.8%) were found in relatively lower proportions. In addition, due to the plethora of molecular docking results, according to reports in the literature, 12 compounds with anti-BC efficacy were selected to plot as a heatmap (Fig. 9b, Table 2). It can be seen from Fig. 9b that 12 compounds with AKT1, EGFR and ESR1 all had good binding activities. We selected the molecular docking results of AKT1 with the main active compounds, and visual analysis was carried out by PyMol software (Fig. 9c). Finally, based on the analysis of molecular docking, SDF were selected in the follow-up in vitro experiment.

**Table 2:** The anti-tumor active substances in RD.

Different types	Com ID	Com name	Antitumor effect	References
Flavonoid	M2	7,4′-Dihydroxyflavone	Inhibiting proliferation	^76^
	M10	Quercetin	Inhibiting proliferation, metastasis and angiogenesis, cell cycle arrest, inducing apoptosis	^72^
	M11	Isorhamnetin	Inhibiting proliferation, inducing apoptosis	^73^
	M13	Liquiritigenin	Inhibiting proliferation and migration, DNA methyltransferase	^25^
	M14	Pinocembrin	Inhibiting proliferation and metastasis	^77^
	M15	7-Hydroxyflavanone	Inhibiting proliferation	^19^
	M22	Isoliquiritigenin	Inhibiting metastasis, neoangiogenesis	^78^
	M27	Loureirin A	Inducing apoptosis	^24^
	M83	Resveratrol	Inhibiting migration and metastasis, inducing apoptosis, cell cycle arrest	^79^
	M84	Pterostilbene	Inhibiting metastasis and proliferation	^80^
Phenolic	M147	Syringaresinol	Inhibiting proliferation	^81^
	M151	Secoisolariciresinol	Inhibiting proliferation	^82^

## Experimental validation in vitro

### SDF inhibited the proliferation of MCF-7 cells

**Figure 10:**
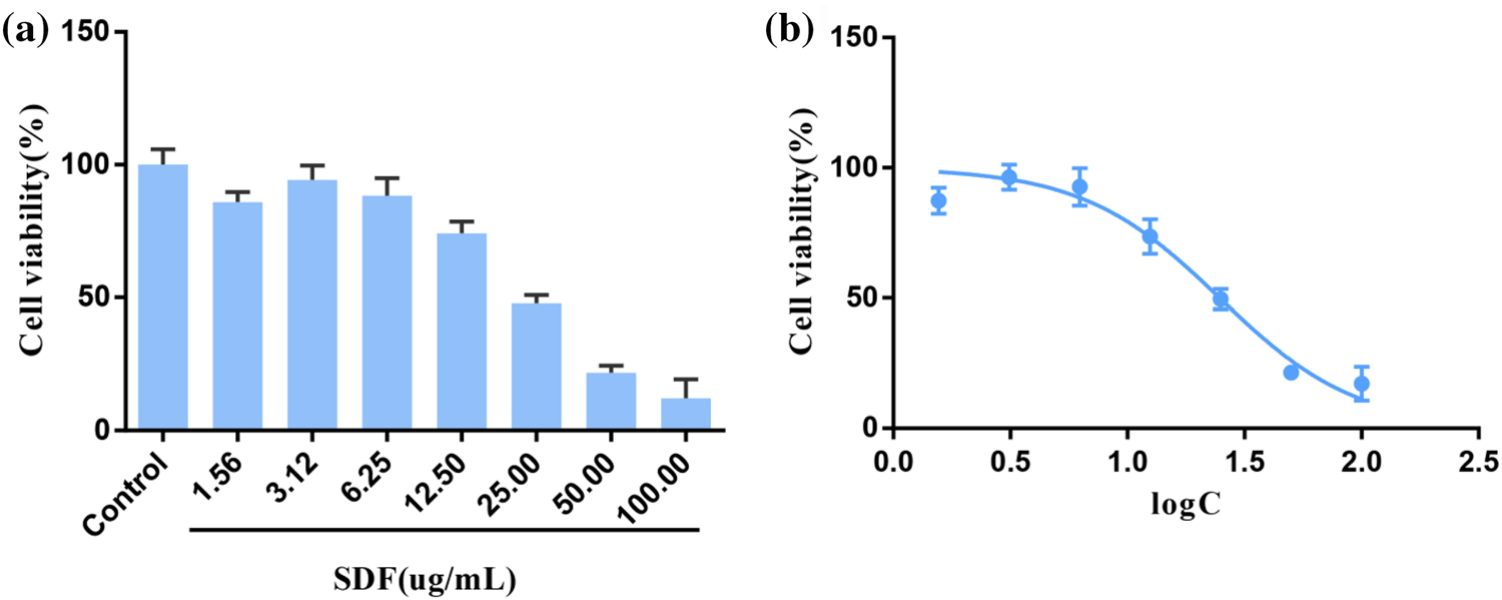
SDF inhibited MCF-7 cell proliferation. (**a**) Cell viability of MCF-7 cells at different concentrations of SDF over 24 h. (**b**) The IC50 value of SDF on MCF-7 cells. The IC50 curve was fitted by cell viability with the drug concentration logarithm.

To verify the antitumor effect of SDF on BC, MCF-7 cells were treated with different concentrations of SDF for 24 h, and cell viability was determined using the CCK-8 assay. Fig. 10a,b show that with increasing concentrations of SDF, MCF-7 cell viability gradually decreased, and the inhibition rate of MCF-7 cells was concentration dependent. The IC50 was 22.68±1.68 µg/mL. This result suggested that SDF can inhibit the proliferation of MCF-7 cells, and thus 10 and 20 μg/mL for 24 h were chosen in the follow-up experiment.

### SDF induced cell apoptosis and cell cycle arrest in MCF-7 cells

**Figure 11:**
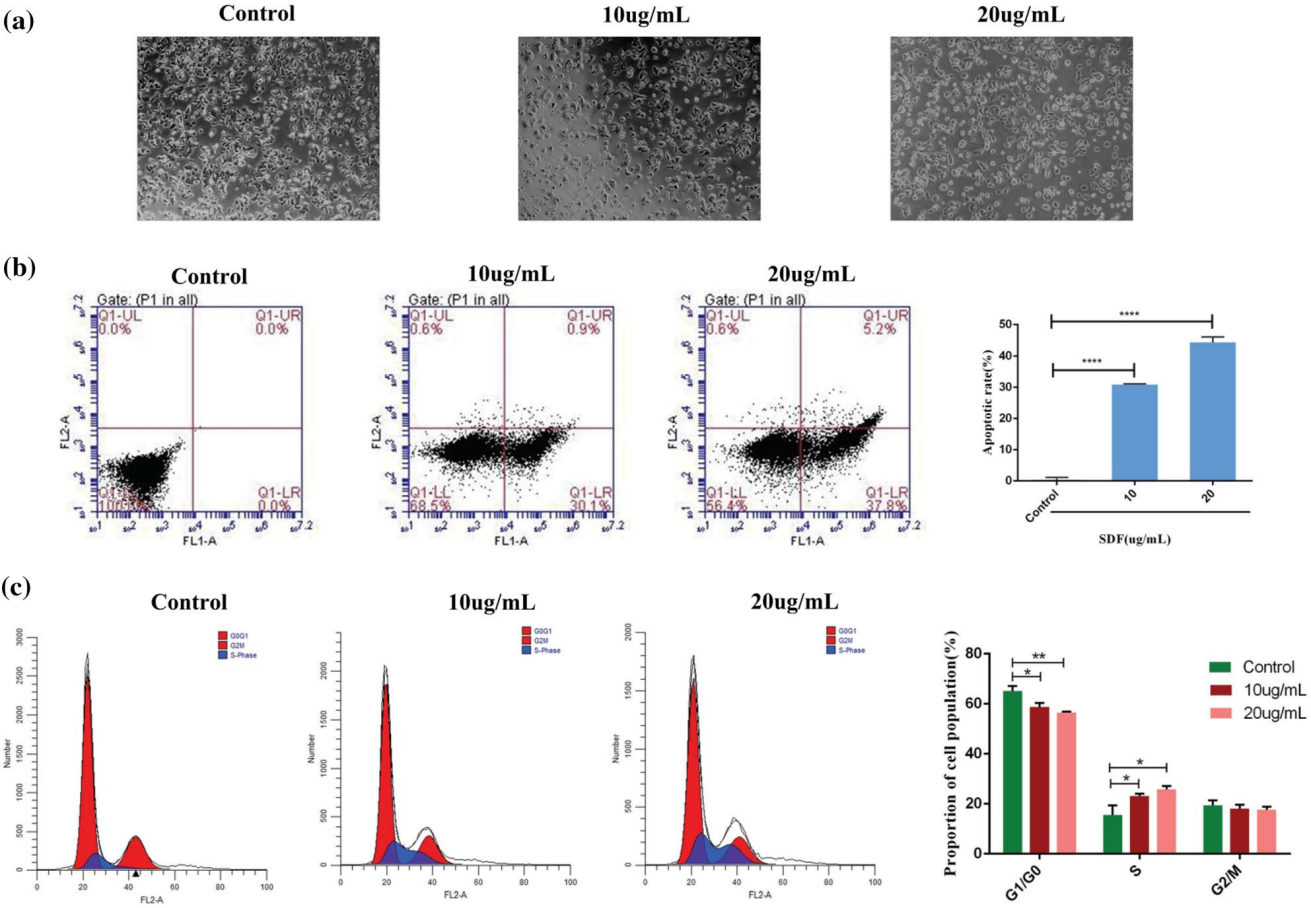
SDF induced cell apoptosis and cell cycle arrest in MCF-7 cells. (**a**) Morphological changes were observed by light microscopy. (**b**) SDF induced MCF-7 cell apoptosis. (**c**) SDF blocked MCF-7 cells in the S phase.

To examine whether SDF induced apoptosis in MCF-7 cells, apoptosis cells were assessed by morphological changes and quantitative analysis. Figure 11a indicates the morphological changes in control and SDF-treated MCF-7 cells observed by light microscopy. Subsequently, an Annexin V/PI staining assay was used to confirm these observations. As shown in Fig. 11b, the percentage of apoptotic cells increased from 30.7 ± 0.25% to 43.3 ± 0.1.75% in MCF-7 cells in a dose-dependent manner. These findings suggested that SDF effectively trigger apoptosis in MCF-7 cells.

Cell cycle arrest is related to both inhibition of proliferation and induction of apoptosis. As shown in Fig. 11c, the increase in the S-phase cell population was accompanied by a corresponding decrease in the G0/G1 and G2/M cell populations. The results showed that SDF arrested MCF-7 cells at the S phase in a dose-dependent manner.

### Effect of SDF on the mRNA expression of 10 hub targets in MCF-7 cells

**Figure 12:**
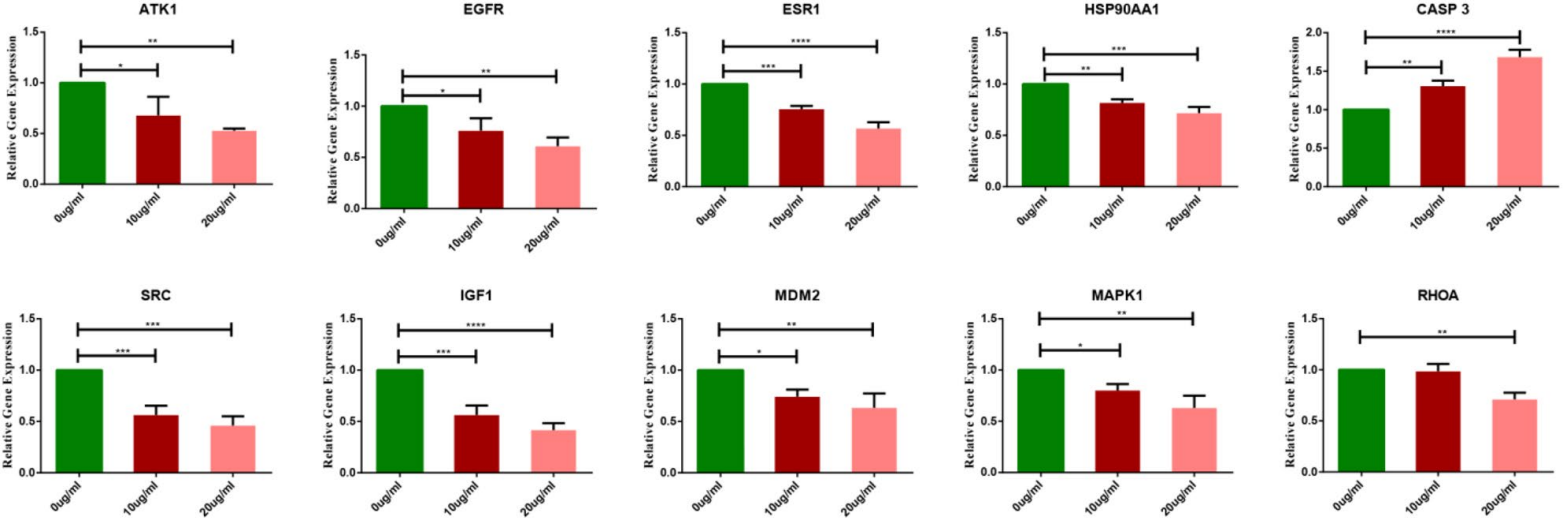
The mRNA expression of 10 hub targets in MCF-7 cells after treatment with SDF (*P < 0.05; **P < 0.01 and ***P < 0.001).

To verify the reliability of the network pharmacology prediction results, the mRNA levels of 10 hub target genes were detected by RT‒PCR in MCF-7 cells. As shown in Fig. 12, the mRNA levels of AKT1, EGFR, ESR1, HSP90AA1, SRC, IGF1, MDM2, MAPK1 and RHOA were significantly downregulated in the SDF-treated groups compared with the controls (P < 0.05), while the mRNA levels of CASP3 and were significantly upregulated (P < 0.05). These results showed that AKT1, EGFR, ESR1, HSP90AA1, SRC, IGF1, MDM2, CASP3, MAPK1 and RHOA are closely related to the BC process as SDF treatment targets.

### Effect of SDF on the PI3K/AKT pathway in MCF-7 cells

**Figure 13:**
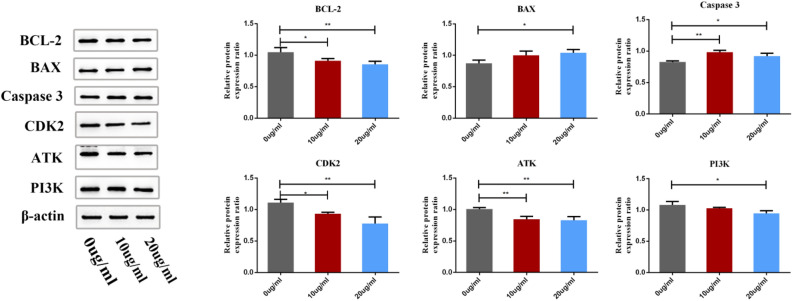
Relative expression of proteins in MCF-7 cells after treatment with SDF (*P < 0.05 and **P < 0.01).

We further explored the molecular mechanism by which SDF induce apoptosis of MCF-7 cells. The PI3K/AKT pathway is likely to be a key pathway associated with the effect of SDF against BC. The protein expression levels of PI3K, AKT, other critical genes enriched in the PI3K/AKT pathway, and BAX were detected by western blotting. As shown in Fig. 13, the protein levels of BCL-2, CDK2, PI3K and AKT were significantly decreased (P < 0.05). In contrast, the protein levels of BAX and Caspase 3 were significantly increased (P < 0.05). The results revealed that SDF can induce apoptosis in MCF-7 cells via the PI3K/AKT pathway.

## Discussion

Currently, surgical resection, radiotherapy, chemotherapy, hormone therapy and targeted therapy are the most common clinical treatment methods for BC; however, surgical resection cannot improve prognosis, and long-term radiotherapy and chemoradiotherapy can cause serious side effects. RD has been widely used clinically to treat a variety of diseases. With further research on RD, its antitumor medicinal value has gradually emerged. In this study, we adopted network pharmacology to explore the molecular mechanism of RD for the treatment of BC; molecular docking and in vitro experiments were further used to verify and provide a theoretical basis for the potential mechanism.

After screening the related datasets, 148 RD target genes were determined. In mechanism exploration, GO analysis showed that multiple targets of RD against BC involved biological processes, including apoptosis, signal transduction, protein phosphorylation, and cell proliferation, thereby suggesting the antitumor mechanism of RD against BC. KEGG analysis indicated that RD is involved in pathways in cancer, the PI3K-Akt signaling pathway, the MAPK signaling pathway, the Ras signaling pathway, and the Rap1 signaling pathway; these pathways have been confirmed to be involved in BC development and progression. The PI3K-Akt signaling pathway is a central signal transduction pathway in BC cell cycle progression and apoptosis and is involved in the targeting mechanism of many new antitumor drugs^30–32^. The MAPK signaling pathway is involved in the processes of cell proliferation, growth, and apoptosis, and plays a crucial role in the BC occurrence and development^33,34^. Reddy et al. found that in MCF-7 cells, lanatoside C can effectively suppress the cell cycle and cell growth by attenuating the MAPK signaling pathway^35^. Rap1, as a central regulator of breast architecture, is activated to induce tumor formation and increase the grade of malignancy^36^. The Rap1 signaling pathway is also involved in breast tumor cell migration and invasion, survival and BC progression^37–39^. Ras is an oncogene whose hyperactivation plays an important role in BC growth and progression, and its effective control is one of the strategies in the treatment of BC^40^. The Ras signaling pathway is involved in cell proliferation and growth, cell survival and apoptosis in BC progression^41,42^. Crosstalk between these signaling pathways could accelerate the growth of BC cells. Hence, according to the results of enrichment analysis, RD could exert its therapeutic effects on BC by regulating the pathways that are closely related to cell proliferation and apoptosis.

Additionally, our predictions from the compound-target-pathway network analysis showed that the cell cycle and key apoptosis genes of RD against BC were enriched in the PI3K-Akt signaling pathway, indicating that RD can exert a major antitumor effect mainly by suppressing the PI3K-Akt signaling pathway. Cell cycle arrest and apoptosis are two key events that regulate cell growth^43^. Hyperactivation of the PI3K/AKT pathway leads to downstream cell survival and inhibition of apoptosis and cell cycle progression. More than 70% of breast tumors have molecular alterations in at least one component of the PI3K/AKT pathway^44^. PI3K activity has been implicated in various tumors, including BC. AKT is the major downstream target of PI3K^45^. AKT regulates cell cycle progression, proliferation and apoptosis through direct phosphorylation^46,47^. Bcl-2 family proteins are crucial mediators downstream of PI3K/Akt signaling^48^. Bcl-2, which is an anti-apoptotic protein, is connected with the overall survival of patients with BC. The balance between Bcl-2 and Bax protein determines cell survival or apoptosis. Caspase 3 is the most important terminal splicing enzyme in apoptosis and triggers the initiation of apoptosis, which is used to treat cancer^49^; for example, tetramethylpyrazine regulates BC cell viability, migration, invasion and apoptosis by repressing Akt signaling and increasing caspase-3 activity^50^. CDK2 is a key regulator of S phase cell cycle progression^51^. Inhibition of CDK2 activity may enhance the potentiated apoptotic effect^52^. Studies have shown that inhibiting AKT and CDK2 activity can suppress BC cell growth^53,54^. In addition, Zhao et al. demonstrated that LYG-202 induces apoptosis and cell cycle arrest by targeting the PI3K/Akt pathway in BC^55^. Based on the above analysis, RD could exert its anti-BC effect by regulating the PI3K/AKT signaling pathway to repress proliferation and induce apoptosis and cell cycle arrest in BC cells.

The 10 hub targets were screened based on the degree after PPI network analysis and then validated in different databases. Numerous studies have reported that the 10 hub targets are closely associated with BC. Activated AKT1 can increase the proliferation and survival of BC cells^56^. The 10 hub targets were screened based on the degree after PPI network analysis and then validated in different databases. Numerous studies have reported that the 10 hub targets are closely associated with BC. Activated AKT1 can increase the proliferation and survival of BC cells^57^. Circulating IGF-1 levels have a positive relationship with BC development^58^. In addition, IGF-1, as a PI3K activator, attenuates LyG-202-induced apoptosis and cell cycle arrest in BC cells^55^. Overexpression of EGFR has been observed in 15%–30% of breast carcinomas and is associated with large tumor size and poor clinical outcomes in BC^59^. Over 80% of BCs are estrogen receptor 1 (ESR1) positive, and its mutations could become a prognostic and predictive biomarker for the treatment of BC^60,61^. CASP3 (Caspase 3) plays an important role in the process of apoptosis, and its high expression is likely to be an independent predictor of BC recurrence^62^. HSP90AA1, which is an intracellular gene, is actively expressed in BC cells; the higher the expression level is, the lower the survival rate^63^. HSP90AA1 is required for efficient cell cycle progression^64^. SRC is a nonreceptor tyrosine kinase that plays key roles in BC development and progression^65^. Silencing SRC causes a reduction in cell migration, attachment, spread and proliferation of MCF-7 cells^66^. MDM2 may play important roles in BC occurrence and development, prognosis, and protection from cancer^67^. MAPK1 is involved in the occurrence, development, metastasis and treatment of BC^67^. Its mutations are associated with many human cancers, including BC^68^. Its mutations are associated with many human cancers, including BC^69^. RhoA is highly expressed in breast malignancies and is closely related to tumor cell proliferation, invasion and metastasis^70^. In addition, validation in different databases showed that the mRNA levels of AKT1, ESR1, HSP90AA1, CASP3, SRC and MDM2 are increased in BC tissues, which may contribute to the occurrence of BC; AKT1, ESR1, CASP3 and SRC may be used as molecular markers in patients with different stages of BC, and ESR1, IGF1 and HSP90AA1 may be used as prospective biomarkers for the diagnosis and prognosis of BC.

Molecular docking results showed that the main active compounds of RD have good binding activity with the hub targets. Among the flavonoid compounds were the key active components that played an anti-BC role by acting on multiple targets (Table 2), including 7,4ʹ-dihydroxyflavone, quercetin, isorhamnetin, liquiritigenin, pinocembrin, 7-hydroxyflavanone, isoliquiritigenin, loureirin A, resveratrol, and pterostilbene. Quercetin can inhibit proliferation of MCF-7 cells by reducing the phosphorylation of P38MAPK^71^. Additionally, quercetin increases the protein expression of proapoptotic Bax and decreases the protein levels of antiapoptotic Bcl-2^72^. Isoliquiritigenin inhibits BC cell proliferation and induces apoptosis by inhibiting the Akt/mTOR and MEK/ERK signaling pathways^73^. Liquiritigenin has a proliferative effect on BC, and as an ERβ agonist, it can repress the invasiveness of the TNBC cell lines HCC1806 and HCC1937^74,75^. For the abovementioned reasons, SDF were selected for subsequent cell experiments to study their potential mechanism.

In vitro experiments showed that SDFcould inhibit MCF-7 cell proliferation, induce cell apoptosis, arrest cells in the S phase, and regulate mRNA expression. The downregulated mRNAs included AKT1, EGFR, ESR1, HSP90AA1, SRC, IGF1, MDM2, MAPK1 and RHOA, and the mRNA expression of CASP3 was upregulated. The results of western blotting implied that BCL-2, CDK2, PI3K and AKT were downregulated, and the protein expression of BAX and Caspase 3 was upregulated, suggesting that SDF may regulate the PI3K/AKT signaling pathway for the treatment of BC. Combined with the above results, SDF significantly inhibited the cell cycle and cell proliferation through the PI3K/AKT pathway and induced apoptosis of MCF-7 cells, consistent with the results predicted by network pharmacological methods.

## Conclusions

In this study, the potential mechanism of RD against BC was initially discussed by using network pharmacology, molecular docking, and in vitro experiments. The results showed that AKT1, ESR1, EGFR, SRC, CASP3, HSP90AA1, IGF1, MDM2, RHOA and MAPK1 were considered as hub targets. Moreover, the active components of RD in BC treatment were composed of 103 compounds, among which flavonoid compounds were identified as the vital active compounds. Some flavonoid compounds of SDF were reported to play a crucial role in the anti-breast cancer process. Experimental further studies provided evidence that SDF can effectively inhibit the proliferation of MCF-7 cells by regulating cell apoptosis and the cell cycle, which may be related to the PI3K/AKT signaling pathway and its targets. Based on this multidisciplinary strategy, the present study provides new insights for future experiments and a valuable theoretical basis of RD in the treatment of BC.

## Materials and methods

### Bioactive compound screening

To gather enough chemical compounds of RD, extensive databases were searched by using the keywords “*dracaena cochinchinensis*”, “dragon's blood”, and “resina draconis”. Then, the structures of the compounds were obtained from PubChem (https://pubchem.ncbi.nlm.nih.gov/), which were used for cross-validation and saved in SDF structure format. The structures were drawn by using ChemDraw 18 if the structure of the compound did not exist in PubChem and saved in SDF structure format. SwissADME (http://www.swissadme.ch/) was used to predict potential active compounds. Compounds with high gastrointestinal absorption (GI) and more than 3 “Yes, 0 violation” in DL analysis were screened out as bioactive compounds for subsequent analysis. The SDF structure data files of bioactive compounds were imported into PharmMapper (http://www.lilab-ecust.cn/pharmmapper/), and compounds without target information were excluded. The official gene symbol format of targets was obtained from the UniProt database (https://www.uniprot.org/), and duplicates were deleted.

### Mining RD target genes for the treatment of BC

BC-related genes were retrieved from DisGeNET (https://www.disgenet.org/), GeneCards (https://www.genecards.org/), Online Mendelian Inheritance in Man (OMIM, https://omim.org/), PharmGKB (https://www.pharmgkb.org/), and Therapeutic Target Database (TTD, http://db.idrblab.net/ttd/) with “breast cancer” as the keyword for searching, all the targets from these databases were merged, and duplicate targets were deleted. The intersection between ingredient targets and disease targets was taken to obtain RD target genes for the treatment of BC, which was visualized by using a Venn diagram (https://www.omicstudio.cn/tool).

### GO and KEGG enrichment analysis

GO and KEGG enrichment analyses were performed using the DAVID database (https://david.ncifcrf.gov/) to determine the potential function of RD target genes. FDR < 0.05 was chosen as the cutoff significance level to screen correlated GO terms and KEGG pathways. The top 20 terms based on the number of enriched genes were plotted at https://www.bioinformatics.com.cn, a free online platform for data analysis and visualization.

### Construction of the compound-target-pathway network

A compound-target-pathway network was built with Cytoscape v3.6.1, of which nodes of different colors represented a compound, target, and pathway, and edges represented compound-target and target-pathway interrelationships.

### Protein‒protein interaction (PPI) analysis and hub target screening

The RD target gene set was input into the STRING database (https://string-db.org/) to obtain PPI data information based on interaction scores (high: > 0.7, medium: > 0.4, low: > 0.15). Then, a PPI interactive network was visualized by using Cytoscape v3.6.1. Finally, the cytoHubba plug-in in Cytoscape v3.6.1 was used to extract the hub genes according to the degree. The top 10 genes were chosen as hub targets.

### Hub gene mRNA expression validation and clinical value analysis

The TCGA database in UALCAN (http://ualcan.path.uab.edu) was used to validate the mRNA expression of the hub genes based on sample type and cancer stage. Significance was considered at a P value of 0.05. The Human Protein Atlas (HPA) (https://www.proteinatlas.org/) was used to explore the protein expression levels of hub targets in normal and BC tissues. The Kaplan‒Meier mapper database (http://kmplot.com/analysis/) was used to analyze the overall survival (OS) value of the mRNA expression of hub targets. The hazard ratio (HR) and log rank P value were calculated and displayed in the graph, and a log rank P value < 0.05 was set as a significant difference. The cBioPortal tool (http://www.cbioportal.org/) was used to discover the genetic information and the correlation between mRNA expression of core targets. A total of 818 breast invasive carcinoma samples (TCGA, Cell 2015) were analyzed. The genomic profiles of 10 hub targets were examined using mutations and putative copy-number alterations in the Genomic Identification of Significant Targets in Cancer (GISTIC) tool.

### Molecular docking validation

Molecular docking was used to verify the binding activity of the hub targets and their corresponding compounds. The PDB IDs corresponding to the hub target proteins selected in this study were AKT1 (PDB ID: 4EJN), ESR1 (PDB ID: 3W2S), EGFR (PDB ID: 4XI3), SRC (PDB ID: 2XJX), CASP3 (PDB ID: 1RE1), HSP90AA1 (PDB ID: 2BDF), IGF1 (PDB ID: 3D94), MDM2 (PDB ID: 3lW8), RHOA (PDB ID: 3lBK), and MAPK1 (PDB ID: 5K4I). The SDF structures of compounds were obtained from PubChem and were converted to the mol2 format using Chem3D software. The PDB format structures of the hub targets were downloaded from the RCSB protein databank (PDB, http://www.rcsb.org/). The solvent molecules and small-molecule ligands were removed by PyMOL software. After preprocessing, structures were generated using AutoDock 1.5.7, and the processed structures were saved in PDBQT format. Molecular docking simulations were performed using AutoDock Vina v1.1.2. to analyze the binding properties (affinity). The affinity was less than -7 kJ∙mol^−1^, which indicated strong binding activity. The results of molecular docking were visualized using the ggplot2 package (v.1.42.0) (http://www.bioconductor.org/) in R (v.3.6.0) language. The combinations of docking scores were visualized by PyMOL2.5.2.

## Experimental verification in vitro

### Preparation of SDF

According to the molecular docking results, SDF were selected as the main effective constituent for experimental verification. SDF powder was purchased from Xishuangbanna Stegosaurus Pharmaceutical Factory. The herbal powder was redissolved in dimethyl sulfoxide (DMSO) (Sigma, USA) at a concentration of 20 mg/mL and then stored at 4 °C for further use.

### Cell cultures and cell viability measurements

The human BC cell line MCF-7 was purchased from Procell Life Science & Technology Co., Ltd. MCF-7 cells were cultured in MCF-7 special culture medium (Procell Life Science & Technology Co., Ltd.) and were incubated at 37 °C under 5% CO_2_ in an incubator. In brief, MCF-7 cells were inoculated into 96-well plates at 1 × 10^4^ cells/well and treated with different concentrations of SDF (100.00, 50.00, 25.00, 12.50, 6.25, 3.12 1.56 and 0 µg/mL) for 24 h. The Cell Counting Kit-8 (CCK-8) (Beyotime, China) assay was used to detect cell proliferation at a wavelength of 450 nm (Molecular Devices SpectraMax i3).

### Apoptosis analysis

An Annexin V Alexa Fluor 488 & Propidium Iodide (PI) Dead Cell Apoptosis kit (Invitrogen, USA) was used to test cell apoptosis according to the manufacturer’s instructions. In brief, MCF-7 cells were incubated in 6-well plates at 37 °C and 5% CO_2_ and treated with SDF at different concentrations (0, 10 and 20 µg/mL) for 24 h. After the treatment, the cells were harvested and washed twice with cold phosphate-buffered saline (PBS), resuspended in 400 μL 1 × binding buffer with 5 μL Annexin V-FITC and 2 μL PI staining solution, and incubated at room temperature in the dark for 20 min. Finally, 100 μL 1 × binding buffer was added, and the cell apoptosis rate was determined by flow cytometry (BD Accuri C6).

### Cell cycle analysis

The Annexin V Alexa Fluor 488 & Propidium Iodide (PI) Dead Cell Apoptosis kit (Invitrogen, USA) was also used to assess the cell cycle. In brief, MCF-7 cells were incubated in 6-well plates at 37 °C and 5% CO_2_ and treated with SDFs at different concentrations (0, 10 and 20 µg/mL) for 24 h. After the treatment, the cells were harvested and washed twice with cold phosphate-buffered saline (PBS) and fixed with 70% ethanol at 4 °C overnight. After centrifugation, the cells were washed twice with cold PBS and resuspended in 400 μL 1 × binding buffer with 2 μL PI staining solution at room temperature in the dark for 20 min. Finally, 100 μL 1 × binding buffer was added and then detected by flow cytometry (BD Accuri C6). The data were analyzed using ModFit 5.0 software.

### Quantitative real-time polymerase chain reaction (qRT‒PCR)

On the basis of network pharmacology results, we selected 10 hub targets to explore the mechanism. Total RNA was extracted by using TRIzol reagent (Beyotime, China) according to the manufacturer’s instructions, and then cDNA was obtained using the BeyoRT™ II First Strand cDNA Synthesis Kit (RNase H-) (Beyotime, China). Quantitative real-time PCR analysis of target gene mRNAs was performed on cDNA using SGExcel UltraSYBR Mixture (with ROX) according to the manufacturer’s instructions (Sangon Biotech, China) and ABI QuantStudio 5. All reactions were run in triplicate. The sequences of the primers for genes are shown in Supplementary Table S9.

### Western blotting assay

To verify the related pathways of drug action on cells, total protein was extracted by using mammalian protein extraction reagent containing 1% protease inhibitor cocktail (CW Biotech, China). The protein concentration was detected using a BCA Protein Quantification Kit (CW Biotech, China), and then the protein was quantified and denatured by adding the protein extraction reagent and loading buffer (CW Biotech). After equal amounts of proteins were loaded onto sodium dodecyl sulfate–polyacrylamide gel electrophoresis (SDS‒PAGE) gels (Biosharp, China), the proteins were transferred to NC membranes (Millipore, Ireland) for 2 h. Then, the membranes were blocked with 5% nonfat milk in Tris-buffered saline Tween 20 (TBST) solution at room temperature for 2 h. The blots were incubated using 5% nonfat milk in TBST solution with the respective primary antibodies against BCL-2, BAX, PI3K, Caspase 3 and β-actin (Abcam, USA) and CDK2 and ATK (Affinity, USA) at 4 °C overnight. After incubation, all membranes were washed three times with TBST solution for 10 min each and incubated with secondary antibodies (CW Biotech, China) at room temperature for 2 h. After incubation, the membranes were washed three times with TBST for 10 min each; the specific protein bands were captured on X-ray film by using enhanced chemiluminescence (CW Biotech, China) and analyzed by densitometry using ImageQuant TL software (GE Healthcare, Uppsala, Sweden).

### Statistical analysis

Statistical analysis was carried out using Prism 6 software. One-way analysis of variance (one-way ANOVA) or Student’s t test was performed on the values, expressed as the mean ± standard deviation (SD) of at least 3 independent experiments. P < 0.05 was defined as significant. In addition, the 50% inhibitory concentration (IC50) value was calculated by using log (inhibitor) versus normalized response—variable slope in nonlinear regression (curve fit).

## Supplementary Information

Supplementary Information 1.

Supplementary Information 2.

Supplementary Information 3.

Supplementary Information 4.

Supplementary Information 5.

Supplementary Information 6.

Supplementary Information 7.

Supplementary Information 8.

Supplementary Information 9.

Supplementary Information 10.

## Data Availability

Data inquiries can be directed to the corresponding author.
