# Supplementary material for: The inhibitory effect and mechanism of Resina Draconis on the proliferation of MCF-7 breast cancer cells: a network pharmacology-based analysis
Source: Sci Rep. 2023 Mar 7;13:3816. doi: 10.1038/s41598-023-30585-0 (PMC9992681; doi:10.1038/s41598-023-30585-0)
Supplement: Supplementary file 1 — Supplementary Information 1. [file 41598_2023_30585_MOESM1_ESM.pdf]

The Inhibitory Effect and Mechanism of Resina Draconis on the Proliferation of MCF-7 Breast Cancer Cells: A Network Pharmacology-Based Analysis

Yana Lv[1,2†] and Yan Mou[3†], Jing Su[1,2], Shifang Liu[1,2], Xuan Ding[1,2],Yin Yuan[1,2] Ge Li[1,2]* and Guang Li[1,2]*

[1] Yunnan Branch of the Institute of Medicinal Plant Development, Chinese Academy of Medical Sciences Jinghong 666100, China
[2] Yunnan Key Laboratory of Southern Medicinal Utilization, Jinghong 666100, China
[3] Yuxi Normal University, Yuxi 653100, China

[†] These authors contributed equally to this work and share first authorship
*** Correspondence Author:**
lige19800021@163.com
gli@implad.ac.cn

Supplementary Information includes:

1. Supplementary Figures S1(this file)

2. Supplementary Tables S1-S9, provided as separate files:

Contents of Tables S1-S9:

Table S1: Chemical compounds isolated from RD.

Table S2: 378 relevant targets of bioactive compounds.

Table S3: 984 BC-related genes.

Table S4: 148 RD target genes for the treatment of BC.

Table S5: 227 Biological processes for RD target genes.

Table S6: 142 KEGG pathways for RD target genes.

Table S7: Component-target-pathway network analysis and evaluation.

Table S8: Binding energy of 10 hub targets and their corresponding 103 active compounds.

Table S9: Sequences of primers for RT-qPCR analyses.

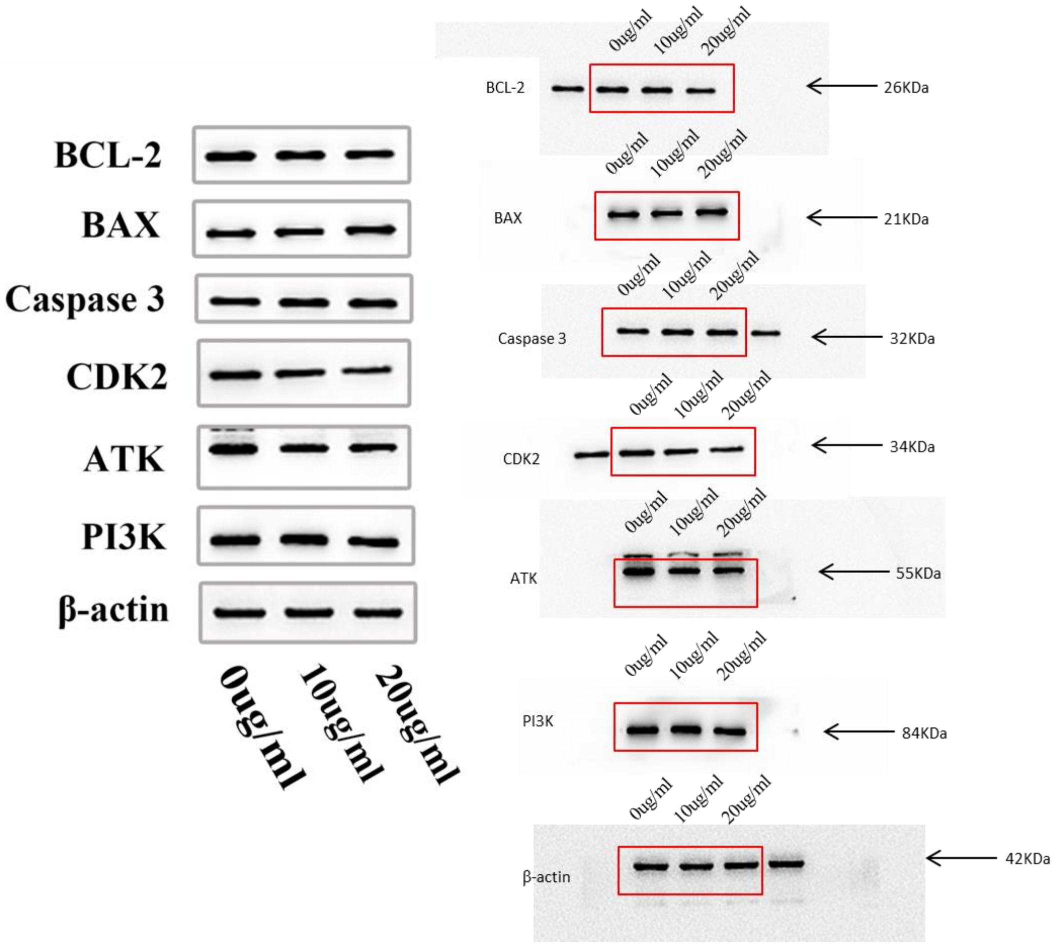

Supplementary FigureS1. Full-length western blots for Figures 13. (Cut the length of NC membrane according to the molecular weight of target protein)
