# Supplementary material for: The inhibitory effect and mechanism of Resina Draconis on the proliferation of MCF-7 breast cancer cells: a network pharmacology-based analysis
Source: Sci Rep. 2023 Mar 7;13:3816. doi: 10.1038/s41598-023-30585-0 (PMC9992681; doi:10.1038/s41598-023-30585-0)
Supplement: Supplementary file 2 — Supplementary Information 2. [file 41598_2023_30585_MOESM2_ESM.docx]

Supplementary Table S1 Chemical Compounds Isolated from RD

| Compound ID | Compound Name | Reference |
| --- | --- | --- |
|  | **Flavonoid Compounds** |  |
| M1 | 7-Hydroxyflavone | (1) |
| M2 | 7,4′-Dihydroxyflavone | (2) |
| M3 | 7,4′-Dihydroxy-5-methoxy-8-methylflavone | (1) |
| M4 | 5,7,4′-Trihydroxyflavone | (1) |
| M5 | 5,7,4′-Trihydroxy-6-methylflavone | (1) |
| M6 | 5,7,4′-Trihydroxy-8-methylflavone | (1) |
| M7 | 7,3′-Dihydroxy-4′-methoylflavone | (3) |
| M8 | 7-Hydroxy-3′-methoy-4′-butoxylflavone | (1) |
| M9 | 6-Methoxy-7-hydroxyflavone | (4) |
| M10 | Quercetin, 3,5,7,3′,4′-pentahydroxyflavone | (5) |
| M11 | Isorhamnetin, 3,5,7,4′-tetrahydroxy-3′-methoxyflavone | (5) |
| M12 | Thevetiaflavone, 7,4′-dihydroxy-5-methoxyflavone | (6) |
| M13 | Glycyrrhizin, liquiritigenin,7,4′-dihydroxyflavanone | (3) |
| M14 | Pinocembrin, 5,7-dihydroxyflavanone | (7) |
| M15 | 7-Hydroxyflavanone | (7) |
| M16 | 5,7,4′-Trihydroxyflavanone | (7) |
| M17 | 7,3′-Dihydroxy-4′-methoxyflavanone | (8) |
| M18 | 7,4′-Dihydroxy-3′-methoxyflavanone | (8) |
| M19 | Echinatin,4,4′-dihydroxy-2-methoxychalcone | (9) |
| M20 | 4,4′-Dihydroxy-2′-methoxychalcone | (3) |
| M21 | 4,4′-Dihydroxy-3′-methoxychalcone | (3) |
| M22 | Isoliquiritigenin, 4,2′,4′-trihydroxychalcone | (10) |
| M23 | 4′-Hydroxy-2′,4-dimethoxychalcane | (11) |
| M24 | 4,4′-Dihydroxy-2′-methoxychalcane | (12) |
| M25 | Cochinchinein A, 4′-hydroxy-2,6-dimethoxydihydrochalcone | (13) |
| M26 | 4′-Hydroxy-4,2′-dimethoxydihydrochalcone | (13) |
| M27 | Loureirin A, 4′-hydroxy-2,4-dimethoxydihydrochalcone | (2) |
| M28 | Loureirin B, 4′-hydroxy-2,4,6-trimethoxydihydrochalcone | (3) |
| M29 | Loureirin C, 4,4′-dihydroxy-2-methoxydihydrochalcone | (3) |
| M30 | 4,4′-Dihydroxy-2,6-dimethoxydihydrochalcone | (3) |
| M31 | 2,4,4′-Trihydroxydihydrochalcone | (14) |
| M32 | Loureirin D, 2,4,4′-trihydroxy-6-methoxydihydrochalcone | (15) |
| M33 | 2,4′-Dihydroxydrochalcone-4-O-β-D-glucoside | (8) |
| M34 | 2,4,2′,5′-Tetrahydroxydihydrochalcone | (8) |
| M35 | 2,4′-Dihydroxy-4-methoxydihydrochalcone | (16) |
| M36 | 6,4′-Dihydroxy-2,4-dimethoxydihydrochalcone | (16) |
| M37 | 4,6,4′-Trihydroxy-2-methoxydihydrochalcone | (17) |
| M38 | 4′-Dihydroxy-2,3′-dimethoxydihydrochalcone | (18) |
| M39 | 4-Hydroxy-2,4′-dimethoxydihydrochalcone | (15) |
| M40 | 3,4′-Dihydroxy-2,4,6-trimethoxydihydrochalcone | (15) |
| M41 | 2,4′-Dihydroxy-4,6-dimethoxydihydrochalcone | (15) |
| M42 | Cochinchinenone, 4-hydroxy-4-[3-(4-hydroxyphenyl)-3-oxopropyl]-3,5-dimethoxycyclohexa-2,5-dienone | (17) |
| M43 | 7,4′-Dihydroxyflavane | (19) |
| M44 | (2S)-7,3′-Dihydroxy-4′-methoxyflavane | (16) |
| M45 | (2S)-7,3′-Dihydroxy-4′-methoxy-8-methylflavane | (20) |
| M46 | (2S)-5,7-Dihydroxy-4′-methoxy-8-methylflavane | (21) |
| M47 | (2S)-5,4′-Dihydroxy-7-methoxy-8-methylflavane | (12) |
| M48 | 7,4′-dihydroxy-8-methylflavane | (3) |
| M49 | (2R)-4′-Dihydroxy-7-methoxy-8-methylflavane | (22) |
| M50 | (±)-7,3′-Dihydroxy-4′-methoxyflavane | (23) |
| M51 | (±)-7,4′Dihydroxy-3′-methoxyflavane | (24) |
| M52 | 7-Hydroxy-4′-methoxyflavane | (2) |
| M53 | 5,4′-Dihydroxy-7-methoxy-6-methylflavane | (3) |
| M54 | 6,4′-Dihydroxy-7-methoxy-8-methylflavane | (9) |
| M55 | 7,4′-Dihydroxy-3′-methoxy-8-methylflavane | (20) |
| M56 | 7,4′-Dihydroxyhomoisoflavane | (2) |
| M57 | 7,4′-Dihydroxy-5-methoxyhomoisoflavane | (15) |
| M58 | 7,4′-Dihydroxy-8-methoxyhomoisoflavane | (25) |
| M59 | Cochinchinenin B,6,4′-dihydroxy-7-methoxyhomoisoflavane | (4) |
| M60 | 7,8-Methylenedioxyphenyl-4′-dihydroxyhomoisoflavane | (9) |
| M61 | 7,3′-Dihydroxy-8,4′-dimethoxyhomoisoflavane | (15) |
| M62 | 4′-Hydroxy-7,8-dimethoxyhomoisoflavane | (15) |
| M63 | 4′-Hydroxy-5,7-dimethoxyhomoisoflavane | (15) |
| M64 | Dracaeconolide B, 7,4′-dihydroxy-5,8-dimethoxyhomoisoflavane | (26) |
| M65 | 6,4′-Dihydroxy-8-methoxyhomoisoflavane | (26) |
| M66 | 7,4′-Dihydroxy-6-methoxyhomoisoflavane | (27) |
| M67 | 5,7,4′-Trihydroxyhomoisoflavane | (27) |
| M68 | 10,11-Tihydroxydracaenone | (3) |
| M69 | 10-Dihydroxy-11-methoxydracaenone | (4) |
| M70 | 7,10-Dihydroxy-4,11-dimethoxydracaenone | (27) |
| M71 | 11-Hydroxy-1,10-dimethoxydracaenone | (27) |
| M72 | 10,11-Dihydroxy-1-methoxydracaenone | (27) |
| M73 | 7,4′-Dihydroxyhomoisoflavanone | (3) |
| M74 | Loureiriol, 3,5,7,4′-tetrahydroxyhomoisoflavanone | (27) |
| M75 | 7-Hydroxy-4′-methoxyhomoisoflavanone | (20) |
| M76 | 3,7-Dihydroxy-8,4′-dimethoxyhomoisoflavanone | (20) |
| M77 | Dracaeconolide A, 3,7-dihydroxy-4′-methoxyhomoisoflavanone | (26) |
| M78 | 7,4′-Dihydroxy-5-methoxyhomoisoflavanone | (27) |
| M79 | 3,7,4′-Trihydroxy-5-methoxyhomoisoflavanone | (27) |
| M80 | 7,4′-Dihydroxyhomoisoflavone | (9) |
| M81 | 5,4′-Dihydroxy-7-methoxyhomoisoflavone | (27) |
| M82 | Trans-pinostilbene, 3,4′-dihydroxy-5-methoxystilbene | (3) |
| M83 | Resveratrol,3,5,4′-trihydroxystilbene | (3) |
| M84 | Pterostilbene, 3,5-dimethoxy-4′-hydroxystilbene | (21) |
| M85 | 3,5-Dihydroxy-4′-methoxystilbene | (17) |
| M86 | Socotrin-4′-ol | (28) |
| M87 | 8-Methylsocotrin-4′-ol | (25) |
| M88 | 2′-Methoxysocotrin-5′-ol | (8) |
| M89 | Cochinchinenin D, 8-methylsocotrin-3′-methoxy-4′-ol | (16) |
| M90 | (2R,γS)-3′-Methoxy-8-methylsocotrin-4′-ol | (28) |
| M91 | (2R,γS)-8-Methylsocotrin-4′-ol | (28) |
| M92 | (2R,γR)-8-Methylsocotrin-4′-ol | (28) |
| M93 | (-)-Cochinchinenin K | (19) |
| M94 | (+)-Cochinchinenin K | (19) |
| M95 | Cochinchinenin L | (19) |
| M96 | Cochinchinenin M | (19) |
| M97 | (2S,γR)-3′-Methoxy-8-methylsocotrin-4′-ol | (19) |
| M98 | cochinchinenin | (3) |
| M99 | Cochinchinenin F | (29) |
| M100 | Cinnabarone | (21) |
| M101 | Cochinchinenin B-1 | (17) |
| M102 | Cochinchinenin C-1 | (17) |
| M103 | 1-[5-(2-Methoxy-4,4′-dihydroxydihydrochalconyl)]-1-(4-hydroxyphenyl)-3-(2-methoxy-4-hydroxyphenyl)propane | (3) |
| M104 | (-)-Cochinchinenin I | (19) |
| M105 | (+)-Cochinchinenin I | (19) |
| M106 | Cochinchinenin E | (29) |
| M107 | Homoisosocotrin-4′-ol | (19) |
| M108 | (-)-Cochinchinenin J | (19) |
| M109 | (+)-Cochinchinenin J | (19) |
| M110 | Cochinchinenene B | (17) |
| M111 | Cochinchinenene C | (17) |
| M112 | Cochinchinenene D | (3) |
| M113 | Cochinchinenene G | (3) |
| M114 | Cochinchinenene A | (3) |
| M115 | Cochinchinenene F | (4) |
| M116 | Cochinchinenene H | (3) |
| M117 | Cochinchinenene E | (4) |
| M118 | Dracaenin A | (29) |
| M119 | Dracaenin B | (29) |
| M120 | Cochinchinenin G | (30) |
| M121 | Cochinchinenin H | (30) |
| M122 | Dracophane | (31) |
| M123 | 10-Hydroxy-11-methoxydracaenone C | (32) |
| M124 | 10,11-Dihydroxydracaenone C | (32) |
| M125 | (3R)-6,4-Dihydroxy-8-methoxyhomoisoflavan | (26) |
| M126 | (3S)-7,4-Dihydroxy-3-(4-hydroxybenzyl)-chromane | (33) |
| M127 | 7,8-Methylenedioxy-4-hydroxyhomoisoflavane | (15) |
| M128 | 7-Hydroxy-3-(4-hydroxybenzyl)-8-methoxychroman | (17) |
| M129 | 3-(4-Hydroxybenzyl)-5,7-dimethoxychroman | (15) |
| M130 | 6-Hydroxy-7-methoxy-3-(4-hydroxybenzyl) chromane | (34) |
| M131 | (3S)-7,4-Dihydroxy-5-methoxyhomoisodihydroflavane | (35) |
| M132 | (±)-5,7,4-Trihydroxy-6-methyldihydrohomoisoflavone | (32) |
| M133 | 7-hydroxy-3-(4-hydroxybenzyl) chroman-4-one | (34) |
| M134 | Cochinchinenins A | (36) |
| M135 | 7-Hydroxy-3-(4-hydroxybenzylidene) chroman-4-one | (32) |
| M136 | Cambodianal | (32) |
| M137 | Apigenin | (35) |
| M138 | 4-Hydroxy-7-methoxy-8-methylflavane | (4) |
| M139 | (2R)-7,4'-Dihydroxyflavan | (33) |
| M140 | (2R)-4'-Hydroxy-7-methoxyflavan | (33) |
| M141 | (2R)-7,4'-Dihydroxy-5-methoxy-8-methylflavan | (26) |
| M142 | 4-Methylcholesta-7-ene-3-ol | (35) |
| M143 | 2-Methoxy-4, 4-dihydroxychalcone | (36) |
| M144 | (3R)-Eucomol | (33) |
|  | Phenolic Compounds |  |
| M145 | 5-Methoxy-2-(4-methoxyphenyl)benzofuran-7-ol | (7) |
| M146 | Cochinchin | (2) |
| M147 | Syringaresinol | (16) |
| M148 | Acanthoside B | (37) |
| M149 | Medioresinol | (16) |
| M150 | 3,3′,5,5′-Tetramethoxy-7′,9-epoxylignan-9′-ol-7-one | (3) |
| M151 | Secoisolariciresinol | (3) |
| M152 | 3,4,5-Trimethoxycinnamyl alcohol | (12) |
| M153 | Dihydrodehydroconiferyl alcohol | (3) |
| M154 | 5-Methoxydihydrodehydroconiferyl alcohol | (3) |
| M155 | (+)-Lyoniresinol | (16) |
| M156 | Dihydrodehydrodiconifery alcohol | (16) |
| M157 | Cochinchinenin C, 1,2,4,5-tetrachloro-dimethoxybenzene | (35) |
| M158 | 4-Hydroxybenzoic ethyl ester | (4) |
| M159 | 1,2,4,5-Tetrachloro-3,6-dimethoxybenzene | (4) |
| M160 | Butylated hydroxytoluene | (38) |
| M161 | 2,4-Di-tert-butylphenol | (38) |
| M162 | 4-Hydroxy-benzaldehyde | (38) |
| M163 | 4-Methylphenol | (38) |
| M164 | 4-Methoxylphenol | (38) |
| M165 | Tachioside | (39) |
| M166 | 4,3,5-Trihydroxystilbene | (33) |
| M167 | 3,4-Dihydroxyallyl benzene | (4) |
|  | **Steroid Compounds** |  |
| M168 | Stigma-5, 22-diene-3-ol | (39) |
| M169 | (25R) spirost-5-en-3β-ol | (4) |
| M170 | Cholest-7-en-3β-ol | (4) |
| M171 | Ergost-8-en-3-ol | (38) |
| M172 | Spirost-5,25(27)-diene-1β,3b-diol | (4) |
| M173 | β-sitosterol | (39) |
| M174 | Diosgenin | (35) |
| M175 | Dracaenosides R | (9) |
| M176 | Dracaenogenin B | (39) |
| M177 | Dracaenol C | (4) |
| M178 | Lophenol | (38) |
| M179 | Brassicasterol | (38) |
| M180 | Cycloartenol | (38) |
| M181 | 26-O-β-d-Glucopyranosyl 25(R,S)-furost-5-en-3,22ξ,26-triol3-O-α-l-rhamnopyranosyl-(1,2)-{β-d-glucopyranosyl(1,3)}-β-d-glucopyranoside | (9) |
| M182 | 26-O-β-d-Glucopyranosyl 25(R,S)-spirost-5-en-3,22ξ,26-triol 3-O-α-l-rhamnopyranosyl-(1,2)-{α-l-rhamnopyranosyl- (1,4)}-β-d-glucopyranoside | (9) |
| M183 | 25(R,S)-dracaenosides M | (9) |
| M184 | 25(R,S)-dracaenosides O | (9) |
| M185 | 25(R,S)-dracaenosides P | (9) |
| M186 | 25(S)-dracaenoside N | (9) |
| M187 | 25(R,S)-spirosta-5-en-3-ol 3-O-α-l-rhamnopyranosyl-(1,2)-β-l-glucopyranosyl-(1,3)]-α-d-glucopyranoside | (9) |
| M188 | 25(R,S)-Spirosta-5-en-3-ol 3-O-α-l-rhamnopyranosyl-(1,2)-{β-d-glucopyranosyl(1,3)}-β-d-glucopyranoside | (9) |
| M189 | 25(R,S)-dracaenosides E | (9) |
| M190 | 25(R,S)-dracaenosides F | (9) |
| M191 | 25(R,S)-dracaenosides G | (40) |
| M192 | 25(R,S)-dracaenosides H | (40) |
| M193 | Dracaenosides I | (40) |
| M194 | Dracaenosides J | (40) |
| M195 | Dracaenosides K | (40) |
| M196 | Dracaenosides L | (40) |
| M197 | Dracaenoside A | (40) |
| M198 | Dracaenoside B | (40) |
| M199 | Dracaenoside C | (40) |
| M200 | Dracaenoside D | (40) |
| M201 | 25(R,S)-dracaenoside Q | (40) |
| M202 | Dracaenogenin A | (39) |
| M203 | Stigmast-5,22-diene-3-O-β-D-glucopyranoside | (4) |
| M204 | (20R, 22S, 25R)-spirost-5-ene-1β,3β,14α,15αtetrol | (35) |
|  | **Other Compound** |  |
| M205 | Heptane | (38) |
| M206 | Octane | (38) |
| M207 | Octadecane | (38) |
| M208 | Undecane | (38) |
| M209 | Tridecane | (38) |
| M210 | Hentriacontane | (38) |
| M211 | 2-hexanone | (38) |
| M212 | Cyclohexane | (38) |
| M213 | Naphthalene | (38) |
| M214 | 2,3-dimethyldecalin | (38) |
| M215 | 2,6-dimethyldecalin | (38) |
| M216 | Decahydro-1,5-dimethylnaphthalene | (38) |
| M217 | Diphenyl sulfide | (38) |
| M218 | τ-cadinol | (38) |
| M219 | τ-muurolon | (38) |
| M220 | α-cadinol | (38) |
| M221 | Dibutyl phthalate | (38) |

**REFERENCES**

1. Peng-Fei TU, Tao J. Flavones from the Wood Dracaena conchinchinensis. *Chin J Nat Med* (2003).

2. Lan H, Zhu HW, Xue HL, De Cai F, Hua ML. Cochinchin from Dracaena cochinchinensis. *Chin J Chem* (2004) 22.

3. Hao Q, Saito Y, Matsuo Y, Li HZ, Tanaka T. Chalcane-stilbene conjugates and oligomeric flavonoids from Chinese Dragon's Blood produced from Dracaena cochinchinensis. *Phytochemistry* (2015) 119: 76-82. doi:10.1016/j.phytochem.2015.09.009

4. Li N, Ma Z, Li M, Xing Y, Hou Y. Natural potential therapeutic agents of neurodegenerative diseases from the traditional herbal medicine Chinese dragon's blood. *J Ethnopharmacol* (2014) 152: 508-21. doi:10.1016/j.jep.2014.01.032

5. Li C, Song QS. Chemical constituents in leaves of Dracaena cochinchinensis. *Chin. Tradit. Herb. Drugs* (2008) 39: 1456-58.

6. Sun J, Song Y, Sun H, Liu W, Zhang Y, Zheng J, et al. Characterization and quantitative analysis of phenolic derivatives in Longxuetongluo Capsule by HPLC-DAD-IT-TOF-MS. *J Pharm Biomed Anal* (2017) 145: 462-72. doi:10.1016/j.jpba.2017.07.012

7. Su X, Li M, Gu Y, Sun J, Zhang J, Huang Z, et al. Phenolic constituents from Draconis Resina. *Tradit. Herb. Drugs* (2014) 45: 1511-14. doi:10.7501/j.issn.0253-2670.2014.11.002

8. Sun J, Liu JN, Fan B, Chen XN, Pang DR, Zheng J, et al. Phenolic constituents, pharmacological activities, quality control, and metabolism of Dracaena species: A review. *J Ethnopharmacol* (2019) 244: 112138. doi:10.1016/j.jep.2019.112138

9. Qing-An Z, Hai-Zhou L, Ying-Jun Z, Chong-Ren Y. Flavonoids from the Resin of Dracaena cochinchinensis. *Helv Chim Acta* (2004) 87.

10. Jiang HM, Wang H, Wang J, Dai HF, Luo YP, Mei WL. [Antibacterial components from artificially induced dragon's blood of Dracaena cambodiana]. *Zhongguo Zhong Yao Za Zhi* (2015) 40: 4002-06.

11. Su XQ, Li MM, Gu YF, Sun J, Zhang J, Huang Z, et al. Phenolic constituents from draconis resina. *Chin. Tradit. Herb.* (2014) 45: 1511-14. doi:10.7501/j.issn.0253-2670.2014.11.002

12. Gonzalez AG, Leon F, Sanchez-Pinto L, Padron JI, Bermejo J. Phenolic compounds of Dragon's blood from Dracaena draco. *J Nat Prod* (2000) 63: 1297-99. doi:10.1021/np000085h

13. Yong KL, Lv JC, Zhang TB, Xu LR, Chen X. A new dihydrochalcone from dragon's blood, red resin of Dracaena cochinchinensis. *Nat Prod Res* (2008) 22: 1624-26. doi:10.1080/14786410701869341

14. Shen CC, Tsai SY, Wei SL, Wang ST, Shieh BJ, Chen CC. Flavonoids isolated from Draconis Resina. *Nat Prod Res* (2007) 21: 377-80. doi:10.1080/14786410701194575

15. Su XQ, Song YL, Zhang J, Huo HX, Huang Z, Zheng J, et al. Dihydrochalcones and homoisoflavanes from the red resin of Dracaena cochinchinensis (Chinese dragon's blood). *Fitoterapia* (2014) 99: 64-71. doi:10.1016/j.fitote.2014.09.006

16. Liu X, Wang B, Gao Y, Rong-Tao LI, Hai-Zhou LI. Phenolic Constituents from Chinese Dragon's Blood and Its Ultraviolet Spectrum Characteristic. *Natural Product Research and Development* (2013).

17. Zhu Y, Zhang P, Yu H, Li J, Wang MW, Zhao W. Anti-Helicobacter pylori and thrombin inhibitory components from Chinese dragon's blood, Dracaena cochinchinensis. *J Nat Prod* (2007) 70: 1570-77. doi:10.1021/np070260v

18. Luo Y, Wang H, Zhao YX, Zeng YB, Shen HY, Dai HF, et al. Cytotoxic and antibacterial flavonoids from dragon's blood of Dracaena cambodiana. *Planta Med* (2011) 77: 2053-56. doi:10.1055/s-0031-1280086

19. Pang DR, Su XQ, Zhu ZX, Sun J, Li YT, Song YL, et al. Flavonoid dimers from the total phenolic extract of Chinese dragon's blood, the red resin of Dracaena cochinchinensis. *Fitoterapia* (2016) 115: 135-41. doi:10.1016/j.fitote.2016.10.004

20. Lin H, Fang-Fang W, Xing-Hong W, Qing-Song Y, Yong X, Wen-Xing L. Phytoconstituents from the leaves of Dracaena cochinchinensis (Lour.) S. C. Chen. *Biochem Syst Ecol* (2015) 63.

21. Chen HQ, Mei WL, Zuo WJ, Wang H, Zhao YX, Dai HF, et al. Chemical constituents from Dragon′ s blood of Dracaena cambodiana. *Chinese Journal of Medicinal Chemistry* (2011) 4: 308-11. doi:10.14142/j.cnki.cn21-1313/r.2011.04.005

22. Ning, Li, Yue, Hou, Mujie, Li, et al. Natural potential therapeutic agents of neurodegenerative diseases from the traditional herbal medicine Chinese dragon's blood. *Journal of Ethnopharmacology: An Interdisciplinary Journal Devoted to Bioscientific Research on Indigenous Drugs* (2014) 152: 508-21.

23. Liu J, Dai H, Wu J, Zeng Y, Mei W. Flavanes from Dracaena cambodiana. *WILEY‐VCH Verlag* (2009).

24. Luo Y, Wang H, Xu X, Mei W, Dai H. Antioxidant phenolic compounds of Dracaena cambodiana. *Molecules* (2010) 15: 8904-14. doi:10.3390/molecules15128904

25. Wang H, Liu J, Wu J. Flavonoids from Dracaena cambodiana. *Chem. Nat. Compd* (2011) 47: 624-26. doi:10.1007/s10600-011-0012-4

26. Xu X, Cheng K, Cheng W, Zhou T, Jiang M, Xu J. Isolation and chatacterization of homoisoflavonoids from Dracaena cochinchinensis and their osteogenic activities in mouse mesenchymal stem cells. *J Pharm Biomed Anal* (2016) 129: 466-72. doi:10.1016/j.jpba.2016.07.017

27. Pang DR, Pan B, Sun J, Sun H, Yao HN, Song YL, et al. Homoisoflavonoid derivatives from the red resin of Dracaena cochinchinensis. *Fitoterapia* (2018) 131: 105-11. doi:10.1016/j.fitote.2018.10.017

28. Guan J, Guo SX. Three new biflavonoids from Chinese dragon's blood, Dracaena cochinchinensis. *Nat Prod Commun* (2012) 7: 591-94.

29. Zheng Q, Xu M, Yang C, Wang D, Li H, Zhu H, et al. A New Red Pigment from Chinese Dragon's Blood, the Red Resin of Dracaena cochinchinensis. *Bull Korean Chem Soc* (2012) 33: 4204-06. doi:10.5012/bkcs.2012.33.12.4204

30. Qing-An Z, Min X, Chong-Ren Y, Dong W, Hai-Zhou L, Hong-Tao Z, et al. Flavonoid oligomers from Chinese dragon’s blood, the red resins of Dracaena cochinchinensis. *Nat Prod Bioprospect* (2012) 2.

31. Vesela D, Marek R, Ubik K, Lunerova K, Sklenar V, Suchy V. Dracophane, a metacyclophane derivative from the resin of Dracaena cinnabari Balf. *Phytochemistry* (2002) 61: 967-70. doi:10.1016/s0031-9422(02)00354-0

32. Wang H, Jiang HM, Li FX, Chen HQ, Liu WC, Ren SZ, et al. Flavonoids from artificially induced dragon's blood of Dracaena cambodiana. *Fitoterapia* (2017) 121: 1-05. doi:10.1016/j.fitote.2017.06.019

33. Likhitwitayawuid K, Sawasdee K, Kirtikara K. Flavonoids and stilbenoids with COX-1 and COX-2 inhibitory activity from Dracaena loureiri. *Planta Med* (2002) 68: 841-43. doi:10.1055/s-2002-34403

34. Gupta D, Bleakley B, Gupta RK. Dragon's blood: Botany, chemistry and therapeutic uses. *Journal of Ethnopharmacology: An Interdisciplinary Journal Devoted to Bioscientific Research on Indigenous Drugs* (2008): 115.

35. Tang Y, Su G, Li N, Li W, Chen G, Chen R, et al. Preventive agents for neurodegenerative diseases from resin of Dracaena cochinchinensis attenuate LPS-induced microglia over-activation. *J Nat Med* (2019) 73: 318-30. doi:10.1007/s11418-018-1266-y

36. Liu X, Chen S, Zhang Y, Zhang F. Modulation of dragon's blood on tetrodotoxin-resistant sodium currents in dorsal root ganglion neurons and identification of its material basis for efficacy. *Sci China C Life Sci* (2006) 49: 274-85. doi:10.1007/s11427-006-0274-4

37. Zhou Z, Wang J, Yang C. Chemical constituents of Sanguis Draxonis made in China. Chin. *Tradit. Herb. Drugs* (2001): 6-08. doi:10.3321/j.issn:0253-2670.2001.06.002

38. Teng Z, Zhang M, Meng S, Dai R, Meng W, Deng Y, et al. A comparative study on volatile metabolites profile of Dracaena cochinchinensis (Lour.) S.C. Chen xylem with and without resin using GC-MS. *Biomed Chromatogr* (2015) 29: 1744-49. doi:10.1002/bmc.3488

39. Zheng QA, Zhang YJ, Yang CR. A new meta-homoisoflavane from the fresh stems of dracaena cochinchinensis. *J Asian Nat Prod Res* (2006) 8: 571-77. doi:10.1080/1028602042000204126

40. Zheng QA, Zhang YJ, Li HZ, Yang CR. Steroidal saponins from fresh stem of Dracaena cochinchinensis. *Steroids* (2004) 69: 111-19. doi:10.1016/j.steroids.2003.11.004
